# LncRNA SNHG11 facilitates tumor metastasis by interacting with and stabilizing HIF-1α

**DOI:** 10.1038/s41388-020-01512-8

**Published:** 2020-10-15

**Authors:** Linguo Xu, Lin Huan, Tianan Guo, Yangjun Wu, Yanfang Liu, Qifeng Wang, Shenglin Huang, Ye Xu, Linhui Liang, Xianghuo He

**Affiliations:** 1 Fudan University Shanghai Cancer Center and Institutes of Biomedical Sciences, Shanghai Medical College, Fudan University, Shanghai, 200032 China; 2 Department of Oncology, Shanghai Medical College, Fudan University, Shanghai, 200032 China; 3 Department of Colorectal Surgery, Fudan University Shanghai Cancer Center, Shanghai, 200032 China; 4 Department of Pathology, Fudan University Shanghai Cancer Center, Shanghai, 200032 China; 5 Key Laboratory of Breast Cancer in Shanghai, Fudan University Shanghai Cancer Center, Fudan University, Shanghai, 200032 China

**Keywords:** Cancer, Cell biology

## Abstract

Epigenetic alteration is one of the hallmarks of colorectal cancer (CRC). Many driver genes are regulated by DNA methylation in CRC. However, the role of DNA methylation regulating lncRNAs remain elusive. Here, we identify that SNHG11 (small nucleolar RNA host gene 11) is upregulated by promotor hypomethylation in CRC and is associated with poor prognosis in CRC patients. SNHG11 can promote CRC cell migration and metastasis under hypoxia. Interestingly, the DNA-binding motif of SNHG11 is similar to that of HIF-1α. In addition, SNHG11-associated genes are enriched with members of the HIF-1 signaling pathway in CRC. Mechanistically, SNHG11 binds to the pVHLrecognition sites on HIF-1α, thus blocking the interaction of pVHL with HIF-1α and preventing its ubiquitination and degradation. Moreover, SNHG11 upregulates the expression of HIF-1α target genes, i.e., AK4, ENO1, HK2, and Twist1. Notably, SNHG11 can bind to the HRE sites in the promoter of these genes and increase their transcription. In summary, these results identify a SNHG11/ HIF-1α axis that plays a pivotal role in tumor invasion and metastasis.

## Introduction

Colorectal cancer (CRC) is one of the most common cancers worldwide and the leading cause of cancer-related deaths [1]. The serious health problem caused by CRC worldwide increases the demand for new biomarkers and therapeutic targets. Epigenetic changes are one of the hallmarks of CRC, particularly DNA methylation alteration. In addition to chromosomal instability and microsatellite instability, a subtype of CRC called CpG island methylator phenotype is identified, which harbors high frequency of DNA hypermethylation [2]. Expression of a group pf genes can be regulated by DNA methylation in the initiation and progression of CRC [3]. Studies on long noncoding RNAs (lncRNAs) have been emerging in the last decade. LncRNAs can regulate the proliferation, apoptosis, invasion, metastasis, and multidrug resistance of CRC, highlighting the potential of lncRNAs to act as new biomarkers and therapeutic targets for CRC patients [4**–**10]. However, whether lncRNAs regulated by DNA methylation play a role in CRC remain elusive. Recently, emerging studies have indicated that RNAs are crucial for the function of transcription factors and chromatin regulators [11**–**14]. The transcription factor Yin-Yang 1 (YY1) can be trapped by RNAs in gene regulatory elements to stabilize the gene expression [15], which suggests that RNAs contribute to the stability of the gene transcription process. Although the binding of RNAs with YY1 might not be strongly sequence specific, YY1 binding sites are enriched in lncRNA-associated enhancer-interacting promoters [16]. In addition to YY1, the chromatin regulator CTCF also interacts with RNAs to regulate chromatin structure [17]. Deletion of RNA binding sites in CTCF significantly dampens the ability of CTCF to form chromatin loops [17]. These findings indicate that the crosstalk between lncRNAs and TF/chromatin regulators is crucial for the chromatin structure and gene transcription program.

To explore the role of DNA-methylation-regulated lncRNAs in CRC, we analyzed DNA-methylation-regulated lncRNAs in CRC cells in previous study, and selected nuclear lncRNAs for further investigation. We found that SNHG11 could promote the invasion and metastasis of CRC cells. Furthermore, we identified that SNHG11 interacted with and stabilized HIF-1α. In addition, SNHG11 enhanced the transcriptional activity of HIF-1α and promoted CRC progression.

## Results

### SNHG11 is upregulated by promoter hypomethylation in CRC

Previous work identified 20 lncRNAs that might be mediated by DNA methylation in The Cancer Genome Atlas (TCGA)-COAD (Supplementary Fig. 1A) [18]. The function of a lncRNA is dependent on its subcellular localization, so knowing the localization of lncRNAs enables the prediction of their biological function [19]. By analyzing RNA-seq data from the lncATLAS database (http://lncatlas.crg.eu/) [20], we determined the subcellular localization of the 18 candidate lncRNAs that were negatively correlated with DNA methylation in TCGA-COAD and found that 7 of them localized in the nucleus (Supplementary Fig. 1B). Among these nuclear lncRNAs, SNHG11 had the basal expression level in nucleus second to PVT1 and had the highest DNA methylation CpG site in normal colon tissues (Supplementary Fig. 1A, B), so we selected SNHG11 for further investigation.

**Fig. 1:**
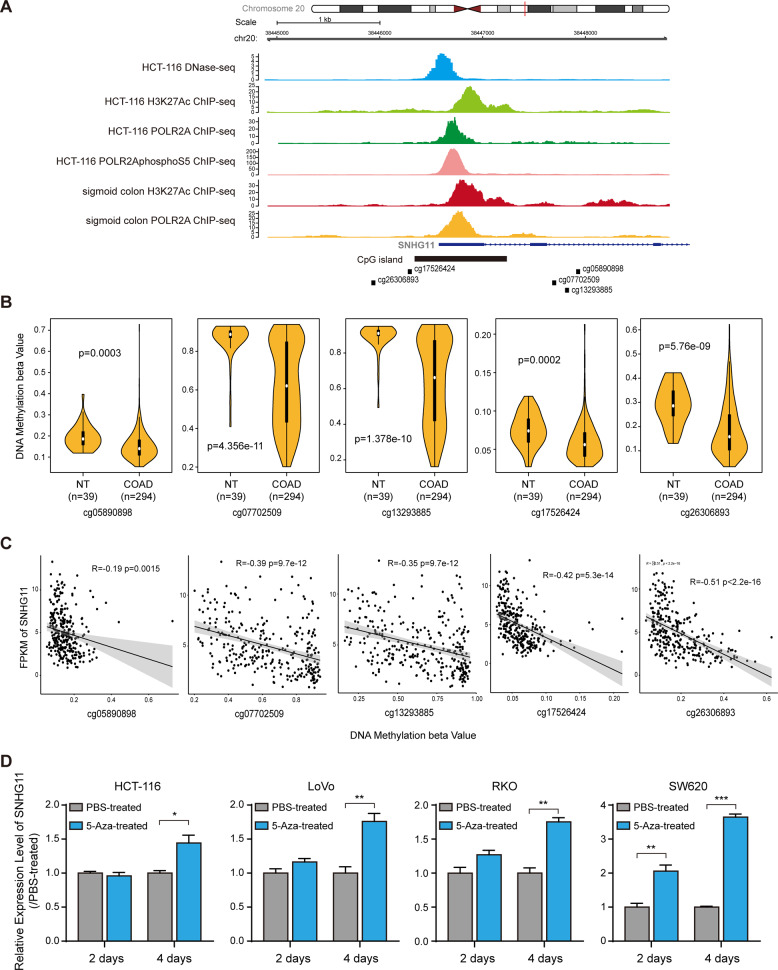
SNHG11 is regulated by DNA methylation. **A** ChIP-seq and DNase-seq data from ENCODE showed the chracteristics of SNHG11 genomic locus. **B** DNA methylation with indicated probes from TCGA-COAD Illumina Human Methylation 450 platform. *n* = 294 CRC tissues and 39 adjacent normal tissues, two-tailed Student’s *t* test. **C** Correlation between DNA methylation and SNHG11 from TCGA-COAD. *n* = 291 CRC tissues in COAD, pearson correlation. **D** SNHG11 expression after 5-Aza treatment was detected by qPCR. *n* = 3 independent experiments, two-tailed Student’s *t* test. *p < 0.05, **p < 0.01, and ***p < 0.001.

We analyzed the characteristics of the genomic locus of SNHG11 and found the CpG island lying at the transcriptional activation site of SNHG11 (Fig. 1A). Interrogating the DNA methylation status from TCGA-COAD, eighteen probes were found covering SNHG11 genomic locus in Illumina Human Methylation 450 platform (Supplementary Fig. 1C). Among these, five sites (cg05890898, cg07702509, cg13293885, cg17526424, and cg26306893) were significantly demethylated in CRC compared to normal tissues (Fig. 1B). In addition, DNA methylation of these sites was negatively correlated with SNHG11 expression in TCGA-COAD (Fig. 1C). These suggested that DNA methylation may modulate SNHG11 expression. Next, we treated CRC cells with 5-Aza, an inhibitor of DNA methyltransferase, and found that the expression of SNHG11 was significantly upregulated in 5-Aza-treated CRC cells (Fig. 1D). Moreover, the induction of SNHG11 in 5-Aza treated cells was consistent with the basal DNA methylation of these cells (Supplementary Fig. 1D).

We performed rapid amplification of cDNA ends (RACE) assays and validated the sequence of SNHG11 (Supplementary Fig. 2A, B), which was located at chromosomal 20q11.23 and consist of five exons (Supplementary Fig. 2B). Next, we determined the subcellular localization of SNHG11 in CRC cells by cellular fractionation and RNA fluorescence in situ hybridization. The results suggested that SNHG11 predominantly localized to the nucleus (Supplementary Fig. 2C, D). The predominant nuclear localization implies a non-protein-coding role for SNHG11, agreeing with the prediction made by the Coding Potential Assessment Tool and by the PhyloCSF score (Supplementary Fig. 2E).

### SNHG11 promotes the migration, invasion, and metastasis of CRC cells in vitro and in vivo

**Fig. 2:**
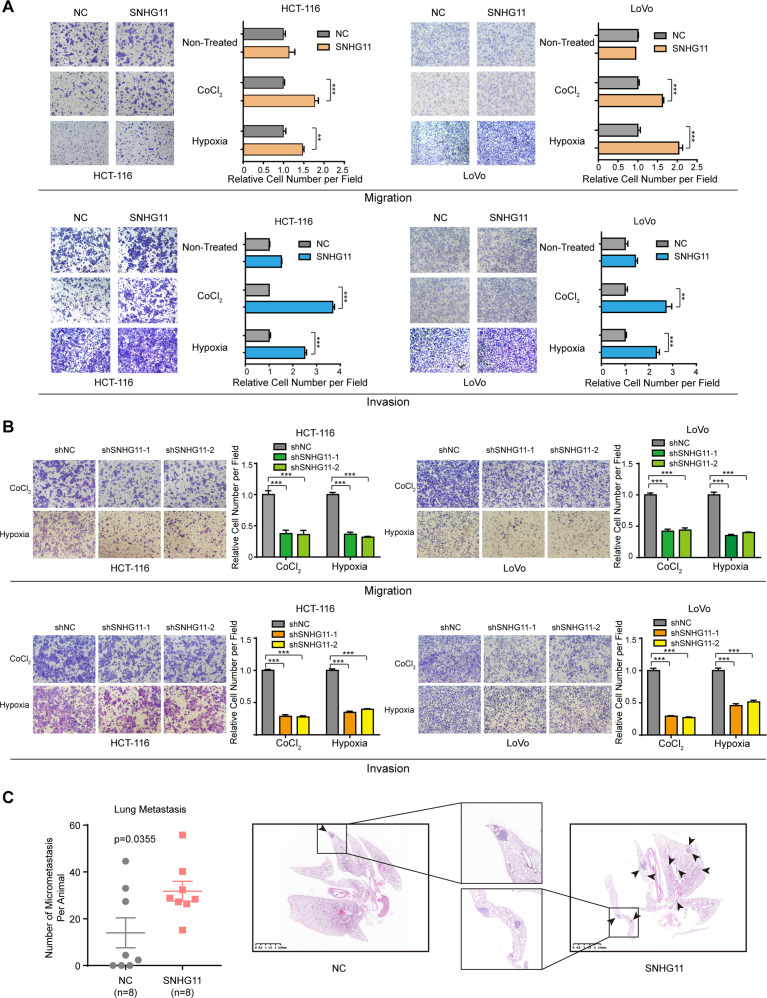
SNHG11 promotes hypoxia-induced CRC cell migration, invasion, and metastasis. **A** Migration and invasion of SNHG11-overexpressing and control CRC cells under non-treated, CoCl_2_-treated, and hypoxic conditions. *n* = 3 independent experiments, two-tailed Student’s *t* test. **B** Cell migration and invasion assays were performed in SNHG11-silenced cells and negative control cells under CoCl_2_-treated and hypoxic conditions. *n* = 3 independent experiments, two-tailed Student’s *t* test. **C** The prometastatic role of SNHG11 was indicated by a mouse model. *n* = 8 mice, two-tailed Student’s *t* test. Representative photographs of pulmonary nodules are shown on the right. *p < 0.05, **p < 0.01, and ***p < 0.001.

To study the potential role of SNHG11 in CRC progression, we performed transwell assays to evaluate the effect of SNHG11 on the migration and invasion of CRC cells. Overexpression of SNHG11 hardly affect the migration and invasion of cells (Fig. 2A and Supplementary Fig. 3A). We hypothesized that SNHG11 might affect CRC cells under external stress, so we performed experiments under hypoxic conditions and found that SNHG11 overexpression significantly increased the migration and invasion abilities of CRC cells treated with CoCl_2_ or 1% O_2_ (Fig. 2A and Supplementary Fig. 3A). Consistently, SNHG11 knockdown suppressed CRC cell migration and invasion under hypoxic conditions (Fig. 2B and Supplementary Fig. 3A). We also performed wound healing assays under normoxic and hypoxic conditions. Consistent with the transwell assays, SNHG11 remarkably increased the wound healing ability of CRC cells treated with CoCl_2_ (Supplementary Fig. 3B, C).

In addition, we further constructed a metastatic lung colonization model by inoculating HCT-116 cells stably transfected with SNHG11 or a control vector into the tail veins of NOD/SCID mice to validate the prometastatic effects of SNHG11 in vivo. The mice were sacrificed 6 weeks later, and the lung tissues were dissected and stained. The results showed that the number of metastatic lung nodules was significantly increased in the SNHG11-overexpressing group compared with that of the control group (Fig. 2C). Collectively, our data suggested that SNHG11 could promote CRC cell invasion and metastasis in vitro and in vivo.

### SNHG11 binds to HIF-1α at its N-terminal pVHL recognition site

**Fig. 3:**
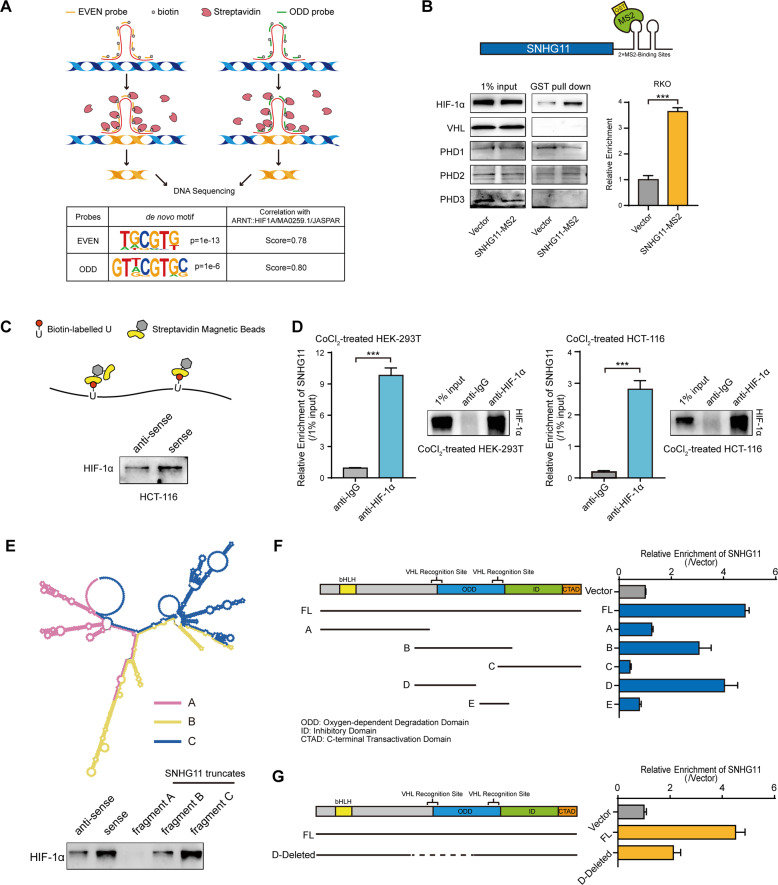
SNHG11 interacts with HIF-1α on the pVHL recognition site of HIF-1α. **A** ChIRP assays of SNHG11 in hypoxia-treated HCT-116 cells. **B** Western blotting following GST pull-down using empty vector or SNHG11-MS2 fusion vector in RKO cells exposed to hypoxia. **C** Western blotting of biotin-labeled RNA pull-down using antisense or sense SNHG11 probes in HCT-116 cells exposed to hypoxia. **D** RIP assays of the enrichment of SNHG11 upon HIF-1α immunoprecipitates in CoCl_2_-treated HEK293T and HCT-116 cells. Anti-IgG was used as a negative control. *n* = 3 independent experiments, two-tailed Student’s *t* test. **E** Western blotting of HIF-1α in biotinylated antisense, full-length or truncated SNHG11 pull-down in HCT-116 cells exposed to hypoxia. **F**, **G** Deletion mapping to identify the SNHG11 binding domain in HIF-1α by RIP-qPCR using full-length or truncated HIF-1α protein. *p < 0.05, **p < 0.01, and ***p < 0.001.

Since SNHG11 is located in the nucleus of CRC cells, we performed chromatin isolation by RNA purification sequencing (ChIRP-seq) to reveal the binding sites of SNHG11 in chromatin. Intriguingly, motif analysis of SNHG11 binding sites using ChIRP-seq data indicated that the SNHG11 binding motif was nearly identical to the HRE sequence based on the HIF-1α motif (Fig. 3A and Supplementary Figs. 4, 5). In addition, SNHG11-correlated genes in CRC were enriched in the HIF-1 signaling pathway (Supplementary Fig. 6A). Gene Set Enrichment Analysis (GSEA) also indicated that SNHG11 is involved in HIF-1α downstream targtes in TCGA-COAD (Supplementary Fig. 6B). Considering that SNHG11 only dramatically promotes CRC cell migration and invasion under hypoxic conditions, we proposed that SNHG11 might interact with HIF-1α or proteins regulating HIF-1α stability. To verify this possibility, we performed an MS2 RNA pull-down assay to evaluate the binding between SNHG11 and HIF-1α, PHDs and pVHL. We found that HIF-1α could bind to SNHG11 (Fig. 3B). Next, we performed RNA pull-down experiments using biotin-labeled SNHG11 and found that SNHG11 interacted with HIF-1α (Fig. 3C). RNA immunoprecipitation (RIP) assay with a HIF-1α antibody indicated that SNHG11 was enriched in HIF-1α immunoprecipitates (Fig. 3D). Moreover, in situ experiments indicated that SNHG11 colocalized with HIF-1α in the nucleus (Supplementary Fig. 6C). These results suggested that SNHG11 bound to HIF-1α. Next, we sought to dissect the interaction between SNHG11 and HIF-1α. We generated a series of SNHG11 truncation mutants and then utilized them in RNA pull-down experiments. Western blot analysis following the RNA pull-down experiments showed that the 601–1064 nt region of SNHG11 was indispensable for the interaction between SNHG11 and HIF-1α (Fig. 3E). Moreover, no increase of migration and invasion was observed in CRC cells overexpressing SNHG11 truncate without 601–1064 nt region under hypoxia (Supplementary Fig. 6D, E).

HIF-1α belongs to the basic helix-loop-helix-PER-ARNT-SIM protein family, and it is regulated by cellular oxygen tension [21]. Under normoxic conditions, PHD enzymes hydroxylate HIF-1α in its oxygen-dependent degradation (ODD) domain [22, 23]. Hydroxylated HIF-1α is recognized, polyubiquitinated, and targeted to the proteasome for degradation by pVHL [24]. To elucidate the binding domain of SNHG11 on HIF-1α, we generated a series of FLAG-tagged HIF-1α domain constructs and transfected these constructs into HEK293T cells. RIP results showed that the constructs including the N-terminal pVHL recognition site could significantly enrich SNHG11 (Fig. 3F and Supplementary Fig. 6F) [24, 25], indicating that the pVHL recognition site might be essential for the interaction with SNHG11. To confirm this, we constructed N-terminal pVHL recognition site-deleted FLAG-tagged expression vectors (Supplementary Fig. 6G). RIP results revealed that deleting the N-terminal pVHL recognition site could significantly decrease the binding of SNHG11 to HIF-1α (Fig. 3G), demonstrating that the N-terminal pVHL recognition site in HIF-1α was the specific binding site for SNHG11.

### SNHG11 stabilizes HIF-1α by blocking the interaction between HIF-1α and pVHL

**Fig. 4:**
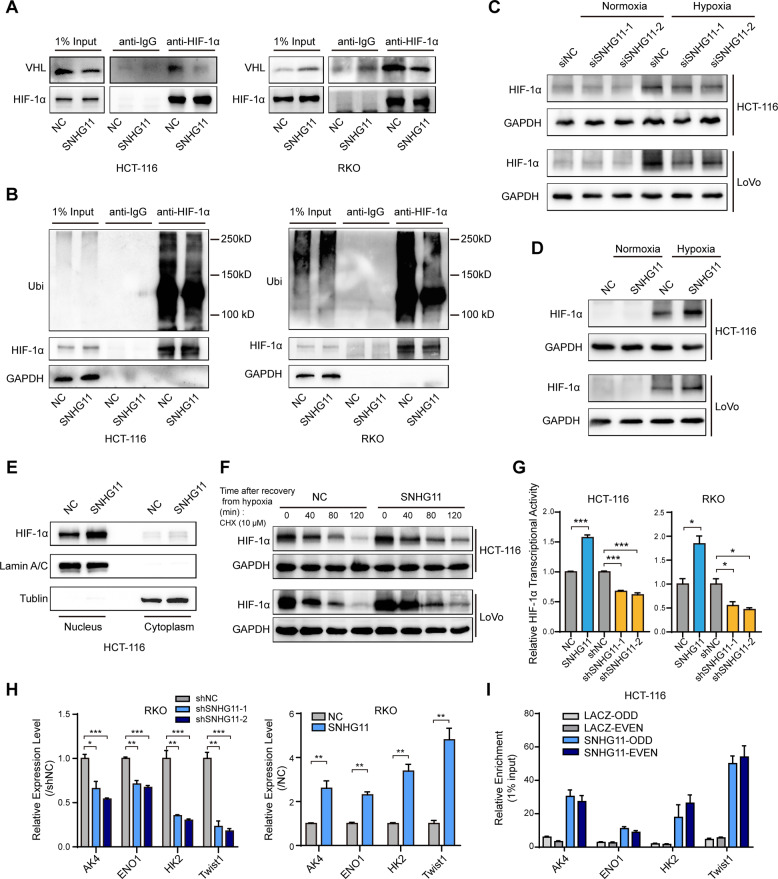
SNHG11 stabilizes HIF-1α by blocking its interaction with pVHL and positively regulates HIF-1α target genes. **A** Immunoprecipitation of anti-HIF-1α in hypoxia- and MG132-treated HCT-116 and RKO cells transfected with SNHG11. **B** The ubiquitination level of HIF-1α in hypoxia- and MG132-treated HCT-116 and RKO cells transfected with SNHG11. **C** The HIF-1α expression level in CRC cells transfected with siSNHG11 and exposed to hypoxia was analyzed by western blotting. **D** The HIF-1α expression level was analyzed by western blotting in CRC cells overexpressing SNHG11 and exposed to hypoxia. **E** Western blotting detected the expression levels of HIF-1α in the cytoplasm and nucleus of HCT-116 cells transfected with SNHG11 and exposed to hypoxia. **F** Control and SNHG11-overexpressing HCT-116 and LoVo cells were incubated under hypoxic conditions for 24 h and then were treated with CHX (10 μM) and were allowed to recover from hypoxia for the indicated times. Western blotting analysis was performed to determine the protein level of HIF-1α. **G** Luciferase reporter assay analysis of HIF-1α transcription activity in CoCl_2_-treated CRC cells after overexpression or knockdown of SNHG11. *n* = 3 independent experiments, two-tailed Student’s *t* test. **H** Expression levels of AK4, ENO1, HK2, and Twist1 in hypoxia-treated CRC cells with SNHG11 overexpression or knockdown. *n* = 3 independent experiments, two-tailed Student’s *t* test. **I** ChIRP-qPCR validated that SNHG11 interacted with HRE sites at AK4, ENO1, HK2, and Twist1 locus in hypoxia-treated HCT-116 cells. *n* = 3 independent experiments, two-tailed Student’s *t* test. *p < 0.05, **p < 0.01, and ***p < 0.001.

Considering that SNHG11 binds to the N-terminal pVHL recognition site of HIF-1α, we next investigated whether SNHG11 could affect the interaction between HIF-1α and pVHL. Our results revealed that SNHG11 overexpression could prevent binding between HIF-1α and pVHL in CRC cells (Fig. 4A and Supplementary Fig. 7A). Following the binding of pVHL and HIF-1α, HIF-1α is ubiquitinated and delivered for proteasomal degradation [22**–**25]. Therefore, we detected the ubiquitination of HIF-1α following SNHG11 overexpression. The results showed that SNHG11 could significantly decrease the ubiquitination level of HIF-1α (Fig. 4B). Next, we tested whether SNHG11 affected HIF-1α expression. The results showed that SNHG11 knockdown reduced HIF-1α protein levels, whereas MG132 treatment diminished this reduction (Fig. 4C and Supplementary Fig. 7B, C). In contrast, SNHG11 overexpression caused an increase in HIF-1α protein levels (Fig. 4D). SNHG11 overexpression under hypoxia did not change pVHL protein levels or HIF-1α mRNA levels (Supplementary Fig. 7D, E). Notably, SNHG11 mainly affected the expression of nuclear HIF-1α (Fig. 4E), which is consistent with the subcellular localization of SNHG11. Since SNHG11 affects the interaction between HIF-1α and pVHL, we studied whether SNHG11 affects HIF-1α stabilization. We treated HTC-116 and LoVo cells with cycloheximide, an inhibitor of protein biosynthesis, after culturing cells in hypoxia. Western blotting analysis showed that SNHG11 overexpression increased the half-life of HIF-1α compared with that of the control cells (Fig. 4F and Supplementary Fig. 7F). Furthermore, the transcriptional activity of HIF-1α was also regulated by SNHG11 (Fig. 4G), suggesting that SNHG11 regulated HIF-1α downstream transcription events under hypoxia. These results demonstrate that SNHG11 not only increases HIF-1α stability by blocking pVHL recognition but also promotes HIF-1α transcriptional activity under hypoxic conditions. Taken together, SNHG11 inhibits the association of pVHL with HIF-1α and alleviates pVHL-mediated ubiquitination of HIF-1α, which causes HIF-1α accumulation.

Next, we selected several genes downstream of HIF-1α that are related to metastasis [26**–**29] and determined their expression by quantitative polymerase chain reaction (qPCR). The results showed that SNHG11 could regulate AK4, ENO1, HK2, and Twist1 expression in CRC cells (Fig. 4H). Based on ChIRP data, we designed primers for HRE sites at these gene promoters and performed ChIRP-qPCR to validate the interaction between SNHG11 and the promoters of these target genes. ChIRP-qPCR showed that SNHG11 bound to HRE sites at the promoters of AK4, ENO1, HK2, and Twist1 (Fig. 4I). Konckdown of these target genes by siRNAs could significantly inhibit the migration and invasion capability of CRC cells under hypoxia (Supplementary Fig. 8A, B). These findings provide evidence that hypoxia-induced SNHG11 increases HIF-1α stability and transcriptional activity by binding to HIF-1α and blocking the interaction between HIF-1α and pVHL.

### The effect of SNHG11 on migration and invasion is mediated by HIF-1α in CRC cells

**Fig. 5:**
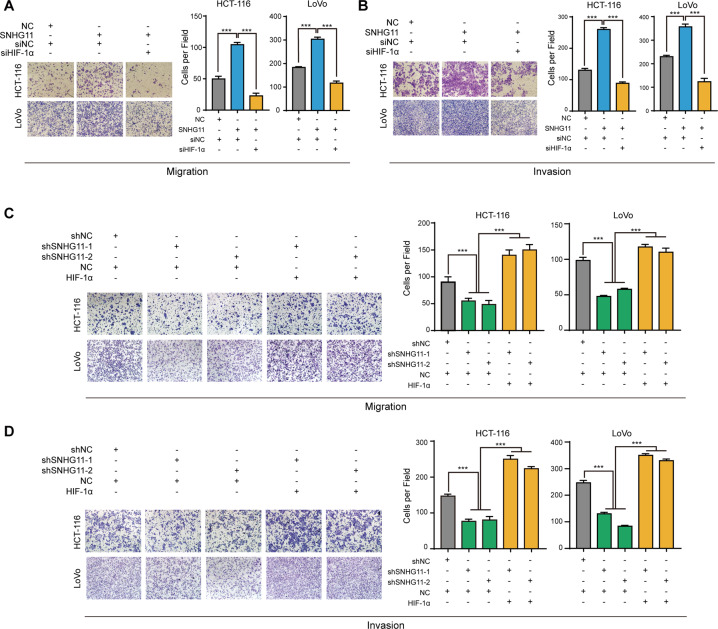
HIF-1α mediates SNHG11-induced cell migration and invasion. **A**–**D** Cell migration and invasion assays of hypoxia-treated HCT-116 and LoVo cells with the indicated treatments. *n* = 3 independent experiments, two-tailed Student’s *t* test. *p < 0.05, **p < 0.01, and ***p < 0.001.

Next, we determined whether SNHG11 exerts its function though HIF-1α. We found that the promotion of SNHG11 on migration and invasion was remarkably abolished when we silenced HIF-1α expression in SNHG11-overexpressing CRC cells (Fig. 5A, B). Consistent with these results, transfecting a HIF-1α plasmid into SNHG11 knockdown cells strikingly eliminated the suppressive influence caused by SNHG11 knockdown on CRC cell migration and invasion (Fig. 5C, D), suggesting that SNHG11-mediated HIF-1α upregulation contributes to the promotion of SNHG11 on CRC cell migration and invasion. Since hypoxia is a common feature of many solid tumors, we explored whether the HIF-1α/SNHG11 axis functions in other cancers. Interestingly, TCGA data revealed that SNHG11 was upregulated in cancers other than CRC (Supplementary Fig. 9A). In addition, SNHG11-correlated genes in rectum adenocarcinoma, prostate adenocarcinoma, kidney renal papillary cell carcinoma, kidney renal clear cell carcinoma, and breast invasive carcinoma were also enriched in the HIF-1 signaling pathway (Supplementary Fig. 9B). These data suggest that the HIF-1α/SNHG11 axis might also function in other cancers.

### SNHG11 is upregulated in CRC and predicts an unfavorable prognosis for CRC patients

**Fig. 6:**
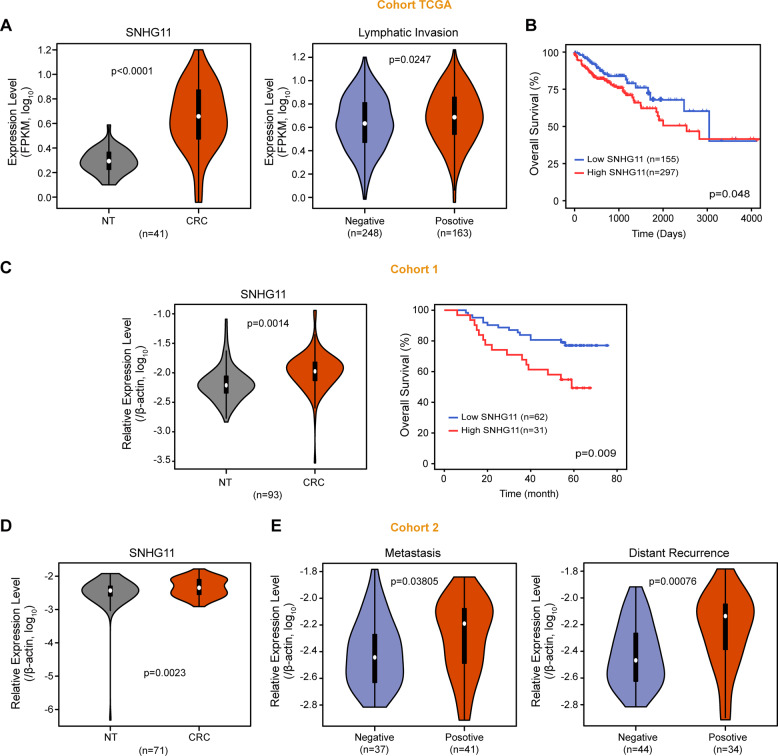
SNHG11 is upregulated in CRC tissues and predicts poor prognosis. **A** SNHG11 expression was increased in 41 paired COAD patients in TCGA (left), and the expression of SNHG11 is shown for COAD patients with or without lymphatic invasion (right) two-tailed Student’s *t* test. **B** Kaplan–Meier analysis of the overall survival curve of CRC patients in COAD-TCGA. **C** The expression and Kaplan–Meier analysis of SNHG11 in 93 paired CRC tissue specimens (cohort 1). *n* = 93 CRC patients, two-tailed Student’s *t* test. **D** The expression of SNHG11 in CRC tissue specimens of cohort 2. *n* = 78 CRC patients and 71 matched adjacent normal tissues, two-tailed Student’s *t* test. **E** The expression of SNHG11 in CRC patients with or without metastasis and distant recurrence, two-tailed Student’s *t* test.

Next, we analyzed the expression and clinical significance of SNHG11 in TCGA database. We found that SNHG11 was frequently upregulated in COAD samples (Fig. 6A). CRC patients with lymphatic invasion had a higher SNHG11 than CRC patients without lymphatic invasion (Fig. 6A). Moreover, CRC patients with higher SNHG11 levels had shorter survival times (Fig. 6B). Then, we examined the expression level of SNHG11 in our independent cohorts 1 (*n* = 93) and 2 (*n* = 78) of CRC samples. Consistent with the results from TCGA data, SNHG11 was significantly upregulated in cohort 1 CRC tissues (Fig. 6C). Moreover, Kaplan–Meier analysis showed that patients with higher SNHG11 expression were associated with reduced overall survival time in cohort 1 CRC samples (Fig. 6C). SNHG11 was also upregulated in cohort 2 CRC samples (Fig. 6D). CRC with metastasis showed higher SNHG11 expression than CRC samples without metastasis (Fig. 6E and Supplementary Table 1). Intriguingly, patients with distant recurrence also had higher SNHG11 levels than patients without distant recurrence (Fig. 6E and Supplementary Table 1). Collectively, these results revealed that SNHG11 was upregulated in CRC and that high expression levels of SNHG11 were associated with poor outcomes in CRC patients.

## Discussion

Most lncRNAs exert their function by binding to various proteins. The cellular distribution of lncRNAs is informative for lncRNA functions [30]. In this study, we analyzed nuclear lncRNAs regulated by DNA methylation and found that SNHG11 regulated the stabilization and transcriptional activity of HIF-1α. Our results indicate that aberrant DNA methylation also regulates the expression of lncRNAs, which can exert important function in CRC progression. Mechanistically, SNHG11 binds to HIF-1α and regulates HIF-1α stability. HIF-1α is upregulated in CRC [31] and exacerbates metastasis by regulating many downstream targets [32]. We discover that SNHG11 promotes the invasion and metastasis of CRC cells. SNHG11 promotes CRC progression by regulating the transcription of HIF-1α downstream targets.

The stabilization of HIF-1α is important for cells in the response to oxygen change. HIF-1α stabilization is tightly regulated by PHD and pVHL. In addition to PHD and pVHL, HIF-1α stabilization can also be regulated by other regulators, including noncoding RNAs. Reports of noncoding RNAs regulating HIF-1α have accumulated in recent years. For example, miR-200b, miR-200c, and miR-429 are induced by HIF-1α and bind to the 3′-UTR of PHD2 mRNA [33]. Overexpression of miR-200 can upregulate HIF-1α protein by downregulating PHD2 protein. Recently, lncRNAs have also been found to be involved in the regulation of HIF-1α stabilization. The cytoplasmic lncRNA LINK-A facilitates the binding between BRK kinase and the EGFR:GPNMB complex and enhances BRK kinase activation, which phosphorylates the HIF-1α protein and stabilizes HIF-1α under normoxic conditions [34]. LncRNAs can also directly bind to HIF-1α protein to stabilize it. LincRNA-p21 disrupts the interaction of HIF-1α and pVHL and promotes hypoxia-induced glycolysis [35]. In our study, we demonstrate that SNHG11 can stabilize and accumulate HIF-1α. SNHG11 binds to the pVHL binding site on HIF-1α and prevents the interaction of HIF-1α with pVHL in the nucleus of CRC cells. Groulx et al. reported that the degradation of HIF-1α can be initiated in the nucleus by PHD prolyl hydroxylation and then can be captured by pVHL [36]; thus, modified HIF-1α is exported to the cytoplasm for proteasome-mediated degradation. Our results reveal that SNHG11 can promote nuclear HIF-1α accumulation under hypoxic conditions. This is different from lincRNA-p21, which stabilizes HIF-1α in the cytoplasm [35]. Our results together with those from previous studies indicate that the regulation of HIF-1α stabilization is complex on multiple layers through various regulators, including noncoding RNAs.

In addition to stabilizing HIF-1α, SNHG11 also increased the transcriptional activation of HIF-1α. SNHG11 promotes the migration and invasion of CRC cells by inducing HIF-1α downstream targets. Shih et al. reported that lncRNA miR31HG can act as a HIF-1α coactivator to drive oral cancer progression [37]. miR31HG is required for hypoxia-induced activation of HIF-1α downstream targets. However, the expression of miR31HG is downregulated in CRC samples [38]. Therefore, we propose that the regulation of HIF-1α by lncRNAs is tissue-dependent. HIF-1α activation requires different lncRNAs in various tissues.

**Fig. 7:**
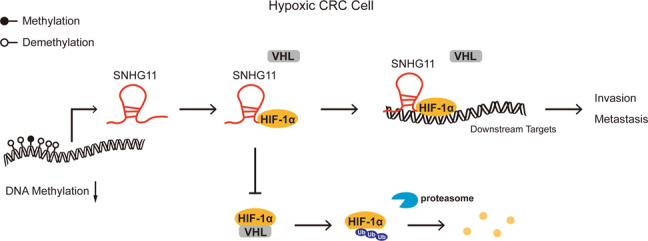
Schematic model of SNHG11-HIF-1α signaling axis in CRC. Demethylated SNHG11 locus increases the expression of SNHG11, and in hypoxic CRC cells SNHG11 interacts with HIF-1α directly and blocks the interaction between HIF-1α and pVHL. As a result, the SNHG11/HIF-1α complex binds to the HRE sites of target genes to promote CRC cell invasion and metastasis.

In conclusion, we demonstrate that lncRNA SNHG11 promotes the stabilization and activation of HIF-1α in CRC cells. SNHG11 promotes CRC progression by reinforcing the invasion and metastasis of CRC cells (Fig. 7). SNHG11 could serve as a prognostic indicator and potential therapeutic target for CRC patients.

## Materials and methods

### Patients and specimens

CRC tissues and adjacent tissues (cohort 1 and cohort 2) were acquired from the surgical specimen archives of Fudan University Shanghai Cancer Center (Shanghai, China). All studies on human specimens were approved by the Ethics Committee of Fudan University Shanghai Cancer Center. No statistical method was used to predetermine sample size due to the availability. Written informed consent was obtained from each patient in accordance with institutional guidelines. Cohort 1 included 93 CRC tissues with matched adjacent normal tissues; Cohort 2 included 78 CRC tissues with 71 matched adjacent normal tissues.

### MS2-based GST pull-down experiments

First, we established RKO cells stably expressing MS2-GST as a fusion protein. SNHG11 was subcloned into a lenti-sgRNA (MS2) vector and named SNHG11-MS2. SNHG11-MS2 or the empty vector was transfected into cells expressing the MS2-GST fusion protein. Cells were then exposed to hypoxia. The experimental procedure was similar to that of the RIP protocol with minor modifications. Briefly, 50 μl of magneGST glutathione particles (Promega, WI, USA) was added to the cell lysate. Twenty percent of the beads were lysed in TRIzol, and 80% of the beads were lysed in SDS loading buffer for Western blotting.

### RNA pull-down assays

Probes of sense, antisense, or truncated SNHG11 RNAs were transcribed and labeled with a Biotin RNA Labeling Mix (Roche, IN, USA); then, they were treated with RNase-free DNase I (TaKaRa, Tokyo, Japan) and were purified with a RNeasy Mini kit (Qiagen, Hilden, Germany). CRC cells treated with hypoxic conditions were lysed in RIPA buffer supplemented with RNase and proteinase inhibitors and then were incubated with equal moles of each biotinylated RNA at 4 °C for 4 h. The mixture was added to 40 μl of prewashed streptavidin beads (Thermo Fisher Scientific, IL, USA) and was incubated on a rotator at room temperature for 1 h. The beads were washed briefly five times with NT2 buffer and boiled in SDS loading buffer. The protein samples were separated by SDS-PAGE. The primers to generate sense, antisense, or truncated SNHG11 sequences are listed in Supplementary Table 2.

### RNA immunoprecipitation (RIP)

Cells were treated with CoCl_2_ for 24 h. RIP experiments were performed with a Magna RIP RNA-Binding Protein Immunoprecipitation kit (Millipore, MA, USA) according to the manufacturer’s instructions. Anti-IgG and the indicated antibodies were used to enrich RNAs. The coprecipitated RNAs were detected by qPCR. For the RIP assays of deletion mutants of HIF-1α, equal plasmids with FLAG-tagged full-length or truncated HIF-1α were transiently transfected into HEK293T cells grown in hypoxic conditions, and cell lysates were immunoprecipitated with a Flag antibody (Sigma-Aldrich, MO, USA).

### In vivo assays

Six-week-old male NOD/SCID mice were housed in laminar flow cabinets under specific pathogen-free conditions with food and water provided ad libitum. Sample size was not predetermined for these experiments. CRC cells (2 × 10^6^) were injected via lateral tail vein injections (*n* = 8 per group). After 8 weeks, the mice were sacrificed, and the lungs were immediately removed. The metastatic nodules were counted after haematoxylin and eosin staining. This study was approved by the Animal Care and Use Committee of Fudan University.

### Chromatin isolation by RNA purification sequencing (ChIRP-seq)

ChIRP assays of SNHG11 in hypoxia-treated HCT-116 cells were performed as previously described [39]. Briefly, we collected CoCl_2_-treated HCT-116 cells and hybridized the sonicated chromatin with biotinylated antisense probe sets against SNHG11 or LacZ (negative control). Probes were grouped into “ODD” and “EVEN” sets based on their positions along the RNA. Then, we purified DNA bound to the probes and performed DNA sequencing. DNA sequencing libraries were constructed from ChIRPed DNA using an NEBNext Ultra II DNA Library Prep kit from Illumina (New England Biolabs, MA, USA). The top 5000 enriched ChIRP sites by EVEN probes or the top 5000 enriched ChIRP sites by ODD probes were used to generate the SNHG11 interacting motifs using the HOMER findmotifsgenome program. The motif length of the findmotifsgenome program was 6, 8, 10, and 12. ChIRP probes are shown in Supplementary Table 3. Peaks generated from ChIRP-seq are shown in Supplementary Table 4.

### Quantitative real-time PCR (qPCR)

RNA was extracted using TRIzol (Invitrogen, CA, USA), and it was reverse transcribed using a PrimeScript RT Reagent kit (TaKaRa, Tokyo, Japan). Real-time qPCRs were performed with SYBR Green Premix Ex Taq (TaKaRa, Tokyo, Japan). Relative RNA expression levels were measured by an ABI system (Thermo Fisher Scientific, IL, USA). The sequences for the gene-specific primers used are listed in Supplementary Table 5. β-actin was employed as an internal control.

### Subcellular fractionation

Cytoplasmic and nuclear fractions were separated by a PARIS cytoplasmic and nuclear extraction kit (Life Technologies, MA, USA). β-actin and U2 were used as cytoplasmic and nuclear positive controls, respectively.

### 5′ and 3′ RACE analysis

5′ and 3′ RACE was performed to determine the transcriptional initiation and termination sites of SNHG11 with a SMARTer RACE cDNA Amplification kit (Clontech, CA, USA) according to the manufacturer’s instructions. The sequences for the gene-specific PCR primers used for 5′ and 3′ RACE analysis are listed in Supplementary Table 6.

### Oligonucleotide transfection

Negative control (siNC) and siRNAs were synthesized by Biotend (Shanghai, China). All siRNAs are shown in Supplementary Table 7. Cells were seeded in six-well plates. The next day, 5 μL of siRNAs and siNC (20 μM) were transfected into cells by Lipofectamine RNAiMAX (Invitrogen, CA, USA) according to the manufacturer’s instructions. Cells were harvested for further experiments after 48 h.

### Lentivirus production and infection

Full-length SNHG11 was generated by RACE experiments and then was cloned into a pCDH-Puro lentiviral vector. The ORF of HIF-1α was cloned into the pCDH-3×Flag lentiviral vector. The primers used to amplify the sequences are listed in Supplementary Table 8. shRNA sequences were synthesized and subcloned into a LentiGuide-Puro lentiviral vector. shRNA sequences are listed in Supplementary Table 8. To generate lentiviruses, pAX2 and pMD2.G were cotransfected into HEK293T cells using Lipofectamine 2000 (Invitrogen, CA, USA). After 48 h, lentiviruses were collected and used to infect CRC cells.

### Western blotting

Proteins were separated by SDS-PAGE and then were transferred to nitrocellulose membranes. The membranes were blocked with 5% milk and probed with primary antibodies overnight at 4 °C. Immune complexes were detected using a LumiBest ECL Reagent Solution kit (Share-Bio, Shanghai, China) after being probed with HRP-conjugated secondary antibodies. The antibodies used in this study are listed in Supplementary Table 9.

### TCGA, ENCODE, and CCLE data

The expression and clinical data of SNHG11 in TCGA-COAD were obtained through the TCGA data portal. Illumina Human Methylation 450 platform-based beta value of DNA methylation in TCGA-COAD was downloaded from Xena (https://xenabrowser.net/). To search the characteristics of the genomic locus, DNase-seq (ENCSR000ENM), H3K27Ac ChIP-seq (ENCSR661KMA), POLR2A ChIP-seq (ENCSR000EUU), POLR2AphosphoS5 ChIP-seq (ENCSR000BML) of HCT-116 cells and H3K27Ac ChIP-seq (ENCSR561YSH), POLR2A ChIP-seq (ENCSR724FCJ) of sigmoid colon were obtained from ENCODE (https://www.encodeproject.org/). DNA methylation status across CRC cell lines was obtained from Cancer Cell Line Encyclopedia (CCLE) (https://portals.broadinstitute.org/ccle/).

Correlations of each genes with SNHG11 in TCGA-COAD were generated from circlncRNAnet (app.cgu.edu.tw/circlnc/circlncRNAnet/) (Supplementary Table 10). GSEA was conducted by the preranked method using the clusterProfiler package in R (https://www.r-project.org/). C2 (curated genesets), C5 (GO genesets), C6 (oncogenic signatures), and hallmark genesets from MSigDB (Molecular Signatures Database) were analyzed.

### Statistical analysis

Data are presented as the mean ± standard error of the mean (SEM) and Student’s *t* test or *χ*^2^-test was analyzed using SPSS 19.0, GraphPad Prism 5, and R project (https://www.r-project.org/). Manuscript investigators were not blinded during the experiments. But there was no human bias given all data performed at least three experiments. Student’s *t* test was performed to evaluate the differences between two groups, followed by F-test to estimate the variation. The Kaplan–Meier method was performed to analyze the correlation between SNHG11 levels and overall survival. Differences with *p* < 0.05 were considered statistically significant (*p < 0.05, **p < 0.01, and ***p < 0.001).

## Supplementary information

Supplementary Information

Supplementary Table 4. Peaks from ChIRP-seq data of SNHG11 under hypoxia

Supplementary Table 10. Correlations of genes with SNHG11 in TCGA-COAD

## Data Availability

The authors declare that all relevant data of this study are available within the article or from the corresponding author on reasonable request.
