# Supplementary material for: LncRNA SNHG11 facilitates tumor metastasis by interacting with and stabilizing HIF-1α
Source: Oncogene. 2020 Oct 15;39(46):7005–18. doi: 10.1038/s41388-020-01512-8 (PMC7661343; doi:10.1038/s41388-020-01512-8)
Supplement: Supplementary file 1 — Supplementary Information [file 41388_2020_1512_MOESM1_ESM.pdf]

# LncRNA SNHG11 Facilitates Tumour Metastasis by Interacting with and Stabilizing HIF-1α

Linguo Xu[1,*], Lin Huan[1,*], Tianan Guo[2], Yangjun Wu[1], Yanfang Liu[1], Qifeng Wang[4], Shenglin Huang[1], Ye Xu[2, #], Linhui Liang[1,3,#], Xianghuo He[1,3,#]

1 Fudan University Shanghai Cancer Center and Institutes of Biomedical Sciences; Department of Oncology, Shanghai Medical College, Fudan University, Shanghai 200032, China

2 Department of Colorectal Surgery, Fudan University Shanghai Cancer Center; Department of Oncology, Shanghai Medical College, Fudan University, Shanghai 200032, China

3 Key Laboratory of Breast Cancer in Shanghai, Fudan University Shanghai Cancer Center, Fudan University, Shanghai 200032, China

4 Department of Pathology, Fudan University Shanghai Cancer Center; Department of Oncology, Shanghai Medical College, Fudan University Shanghai 200032, China

* These authors contributed equally to this work

[#] Correspondence: Xianghuo He, Ph.D., xhhe@fudan.edu.cn, or Linhui Liang, PhD., lianglinhui@fudan.edu.cn, Fudan University Shanghai Cancer Center and Institutes of Biomedical Sciences, Shanghai Medical College, Fudan University; or Ye Xu, MD, xuye021@163.com, Department of Colorectal Surgery, Fudan University Shanghai Cancer Center. 302 Rm., 7# Bldg., 270 Dong An Rd., Shanghai 200032, China. Tel.: 86-21-34777580; Fax: 86-21-64172585.

# Supplementary Information

## Supplementary Materials and Methods

Cell culture
Cell wound healing assay
Migration and invasion assays
Fluorescence *in situ* hybridization
Co-immunoprecipitation

## Supplementary Figures

Supplementary Fig 1. DNA Methylation of SNHG11.
Supplementary Fig 2. Characteristic features of SNHG11.
Supplementary Fig 3. SNHG11 facilitates the wound healing ability of $CoCl_2$-treated CRC cells.
Supplementary Fig 4. Motif analysis of ChIRP-seq of SNHG11 by EVEN probes.
Supplementary Fig 5. Motif analysis of ChIRP-seq of SNHG11 by ODD probes.
Supplementary Fig 6. SNHG11 interacts with HIF-1α in CRC cells.
Supplementary Fig 7. SNHG11 stabilizes HIF-1α in CRC cells.
Supplementary Fig 8. Downstream targets of SNHG11/HIF-1α are involved in migration and invasion of CRC cells under hypoxia.
Supplementary Fig 9. SNHG11/HIF-1α may exert function in other cancers.

## Supplementary Tables

Supplementary Table 1. Association between SNHG11 expression and clinicopathological variables of the studied CRC patients.
Supplementary Table 2. Sequences of primers for RNA pull down experiments.
Supplementary Table 3. Sequences of ChIRP probes.
Supplementary Table 4. Peaks from ChIRP-seq data of SNHG11 under hypoxia.
Supplementary Table 5. Sequences of qPCR primers to detect RNA expression.
Supplementary Table 6. Sequences of primers in RACE.
Supplementary Table 7. Sequences of siRNAs used in this study.
Supplementary Table 8. Sequences of primers used in this study.
Supplementary Table 9. Antibodies used in this study.
Supplementary Table 10. Correlations of genes with SNHG11 in TCGA-COAD.

# Supplementary Materials and Methods

## Cell culture

Human CRC cell lines (HCT-116, LoVo and RKO) and HEK293T cells were purchased from the American Type Culture Collection (ATCC, USA). Cell lines were authenticated by STR profiling and confirmed to be mycoplasma-free. HCT-116 cells were cultured in McCoy's 5A medium, Lovo cells were cultured in Ham's F-12K medium, RKO cells were cultured in RPMI-1640 medium and HEK293T cells were cultured in DMEM medium at 37°C in 95% air and 5% $CO_2$. All media were supplemented with 10% foetal bovine serum, 100 U/ml penicillin and 100 U/ml streptomycin. 10 μM 5-Aza-2′-deoxycytidine (Sigma-Aldrich, MO, USA) was used to inhibit DNA methylation in CRC cells for indicated days. For hypoxia induction, cells were incubated in a hypoxic chamber with a gas mixture of 94% $N_2$/5% $CO_2$/1% $O_2$. The cells were incubated for 24h under hypoxic conditions.

## Cell wound healing assay

RKO cells were seeded in 12-well plates and were cultured until they were 100% confluent. A scratch was created by scratching cell monolayers with a sterile 200 μl plastic pipette tip. Cells were then incubated with or without $CoCl_2$ for 12 h. Images were captured by phase contrast microscopy.

## Migration and invasion assays

Migration and invasion assays were performed using a 24-well transwell chamber (Corning, NY, USA). Briefly, CRC cells ($5\times10^4$ in 200 μl serum-free medium) were seeded into the upper chamber. The lower chamber was filled with medium containing 10% foetal bovine serum for 24 h with or without exposure to hypoxic conditions. Cells on the upper membrane surface were wiped off using a cotton swab. Migrated cells attached to the lower membrane surface were fixed with methanol and stained with crystal violet. For invasion assays, the upper chamber was precoated with Matrigel.

## Fluorescence *in situ* hybridization

CRC cells were seeded on sterile glass coverslips and incubated with $CoCl_2$ for 24h. Then, the cells were fixed with 4% PFA for 10 min. Probes designed for ChIRP were used to detect SNHG11 and were incubated with cells for 1 h at 37°C. After brief washing with saline-sodium citrate, cells were incubated with an anti-HIF-1α antibody (Proteintech, IL, USA). After secondary antibody incubation, tyramide signal amplification (Thermo Fisher Scientific, IL, USA) and DAPI staining, images were captured by confocal microscopy (Leica, Germany).

## Co-immunoprecipitation

Cells grown in dishes were exposed to hypoxic conditions for 24 h and then were treated with MG-132 (Selleck, Shanghai, China) for 12 h before being lysed in IP lysis buffer (25 mM Tris-HCl pH 7.4, 150 mM NaCl, 1 mM EDTA, 1% NP-40 and 5% glycerol) supplemented with RNase and proteinase inhibitors. Anti-IgG and anti-HIF-1α were incubated with Protein A/G magnetic beads (Bimake, Shanghai, China) at room temperature for 30 min. Equal volumes of cell lysate supernatant were added to anti-IgG or the indicated antibody-labelled beads and incubated overnight at 4°C. The next day, beads were washed five times with NT2 buffer and then were boiled in SDS loading buffer.

# Supplementary Figures

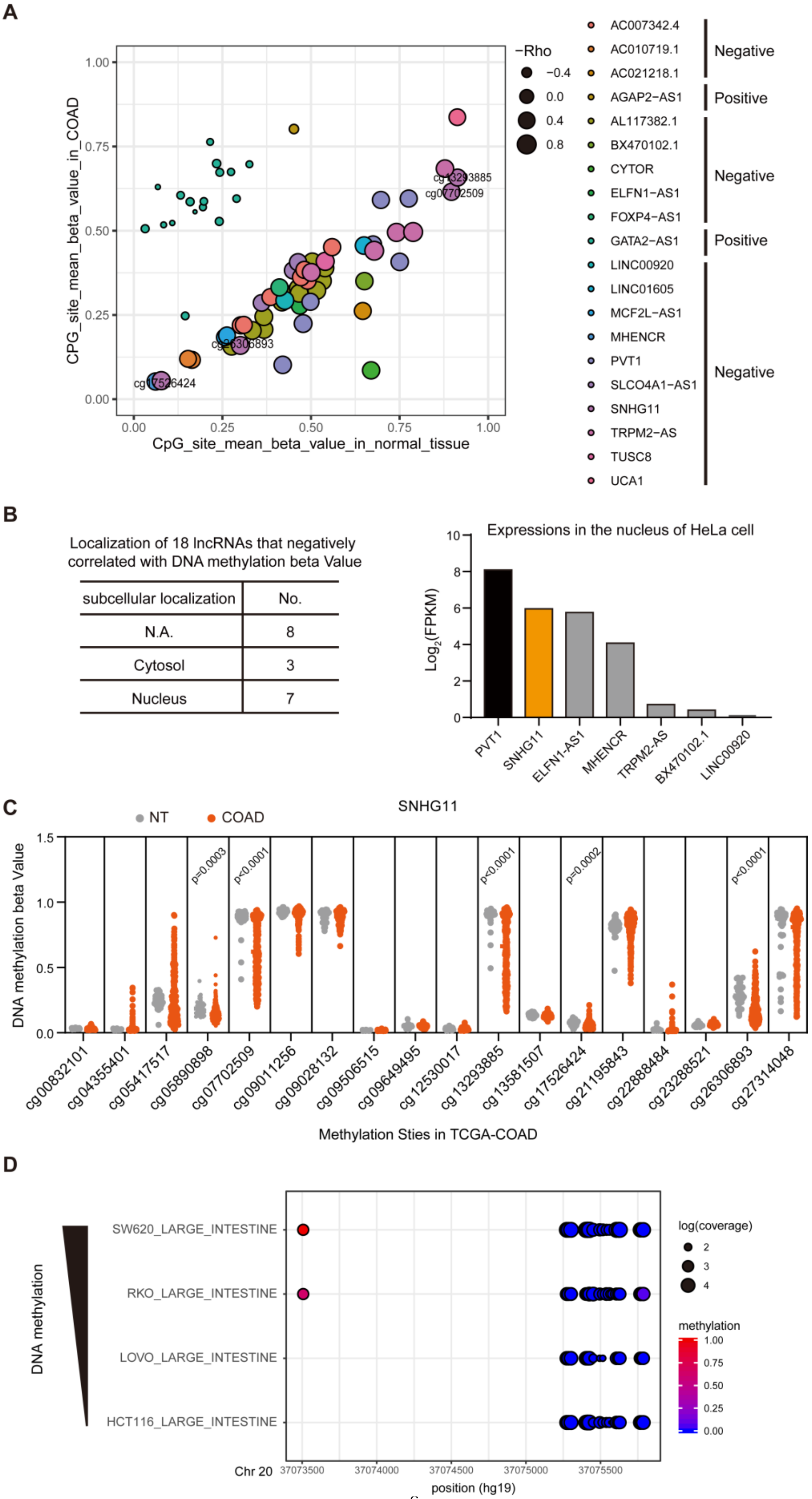

**Supplementary Fig 1. DNA Methylation of SNHG11.** (A) CpG site methylation of 20 lncRNAs in TCGA COAD. (B) Distribution of lncRNAs in subcellular localization and expression level of lncRNA in the HeLa nuclear localization. (C) DNA methylation across SNHG11 locus from TCGA-COAD. Platform: Illumina Human Methylation 450. n=39 normal tissues and 294 CRC tissues in COAD, two-tailed Student's t-test. (D) DNA methylation across SNHG11 loci in CRC cells were obtained from CCLE database.

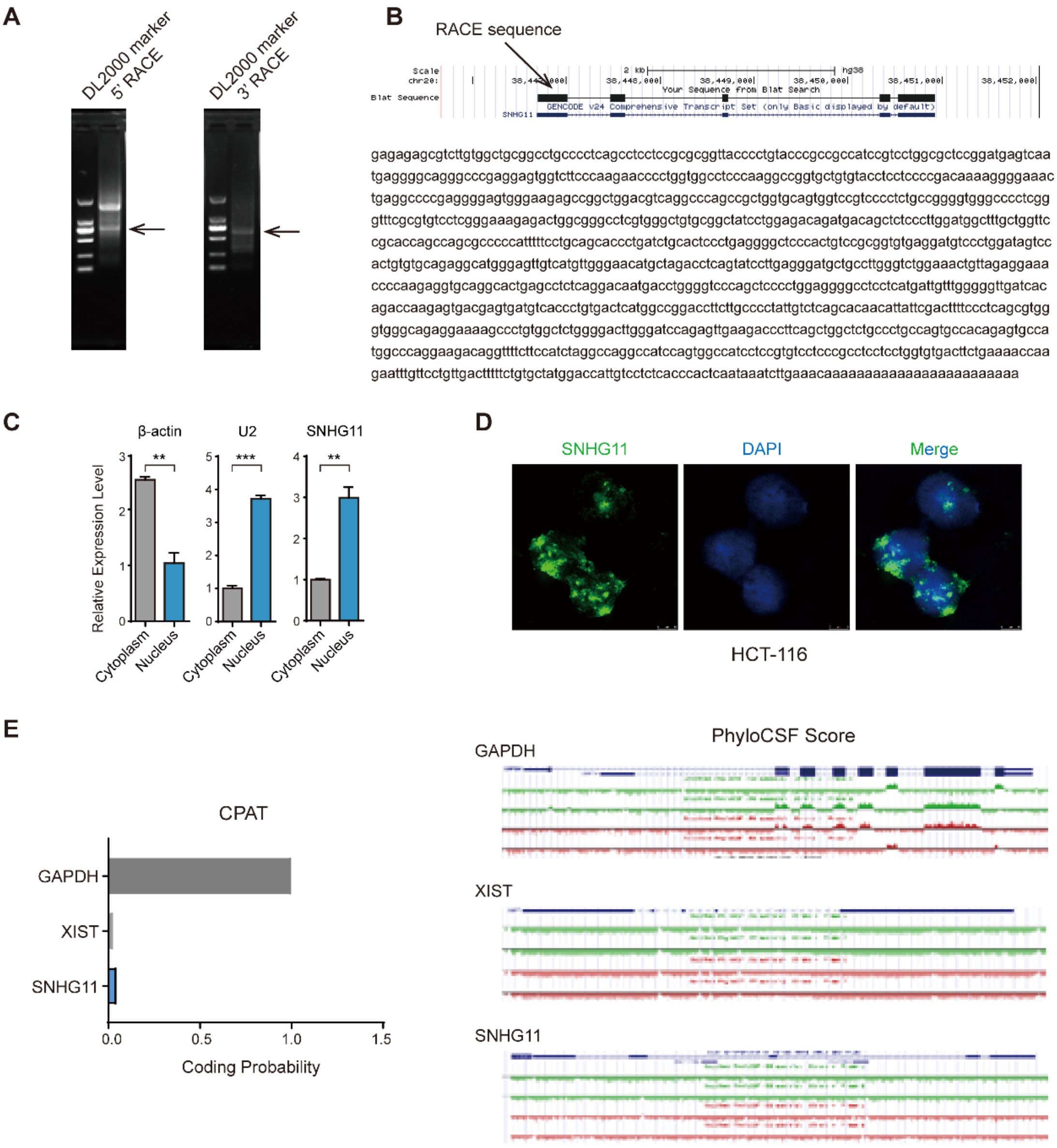


**Supplementary Fig 2. Characteristic features of SNHG11.** (A and B) 5’ and 3’ RACE assays of SNHG11. (C) Nuclear/cytoplasmic RNA fractionation analysis of the location of SNHG11 in HCT-116 cells. n=3 independent experiments, two-tailed Student’s t-test. (D) Confocal microscopic images of SNHG11 in HCT-116 cells treated with hypoxia. (E) Coding Potential Assessment Tool (CPAT, http://lilab.research.bcm.edu/cpat/) and PhyloCSF to estimate the coding probability of SNHG11. * $p < 0.05$, ** $p < 0.01$, and *** $p < 0.001$.

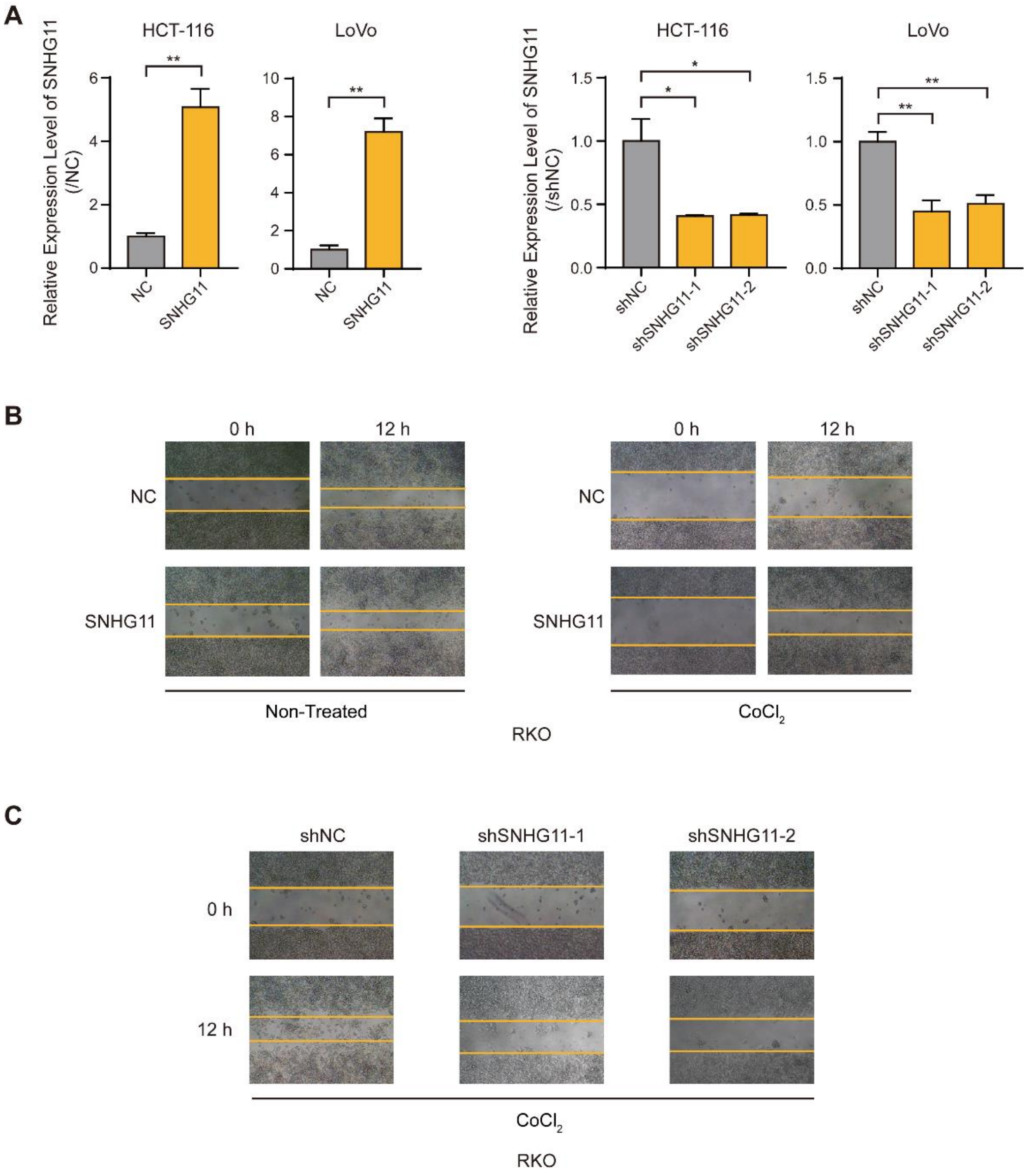


**Supplementary Fig 3. SNHG11 facilitates the wound healing ability of $CoCl_2$-treated CRC cells.** (A) Overexpression and knockdown efficiency of SNHG11 in CRC cells. n=3 independent experiments, two-tailed Student's t-test. (B and C) Wound healing assays of CRC cells with indicated treatment. * $p < 0.05$, ** $p < 0.01$, and *** $p < 0.001$.

## Homer de novo Motif Results of EVEN probes

| Rank | Motif | P-value | log P-pvalue | % of Targets | % of Background | STD(Bg STD) | Best Match/Details | Motif File |
|---|---|---|---|---|---|---|---|---|
| 1 | GTCCTCTCACCC | 1e-106 | -2.459e+02 | 4.34% | 0.45% | 48.5bp (53.4bp) | NR2C2/MA0504.1/Jaspar(0.712) More Information \| Similar Motifs Found | motif file (matrix) |
| 2 | ACACGCGAAACC | 1e-44 | -1.020e+02 | 1.89% | 0.21% | 57.0bp (63.4bp) | IRF4(IRF)/GM12878-IRF4-ChIP-Seq(GSE32465)/Homer(0.687) More Information \| Similar Motifs Found | motif file (matrix) |
| 3 | TCGCGTGTCCTC | 1e-33 | -7.753e+01 | 1.29% | 0.13% | 52.2bp (55.3bp) | PAX5/MA0014.3/Jaspar(0.715) More Information \| Similar Motifs Found | motif file (matrix) |
| 4 | TGTCCTCCACCC | 1e-28 | -6.624e+01 | 1.61% | 0.27% | 46.3bp (54.7bp) | SMAD2::SMAD3::SMAD4/MA0513.1/Jaspar(0.670) More Information \| Similar Motifs Found | motif file (matrix) |
| 5 | GGACAATGGT | 1e-26 | -6.000e+01 | 2.33% | 0.61% | 53.4bp (57.2bp) | SOX13/MA1120.1/Jaspar(0.819) More Information \| Similar Motifs Found | motif file (matrix) |
| 6 | TGCACACAGTGG | 1e-23 | -5.410e+01 | 0.37% | 0.01% | 38.2bp (65.9bp) | Oct4:Sox17(POU,Homeobox,HMG)/F9-Sox17-ChIP-Seq(GSE44553)/Homer(0.710) More Information \| Similar Motifs Found | motif file (matrix) |
| 7 | GGTGAGCGTGTC | 1e-23 | -5.410e+01 | 0.37% | 0.01% | 60.4bp (50.9bp) | Klf9(Zf)/GBM-Klf9-ChIP-Seq(GSE62211)/Homer(0.713) More Information \| Similar Motifs Found | motif file (matrix) |
| 8 | AACTCTGGATCC | 1e-18 | -4.171e+01 | 0.67% | 0.07% | 45.0bp (48.7bp) | ZBTB12(Zf)/HEK293-ZBTB12.GFP-ChIP-Seq(GSE58341)/Homer(0.605) More Information \| Similar Motifs Found | motif file (matrix) |
| 9 | GTTTGGCGGTCT | 1e-16 | -3.795e+01 | 0.37% | 0.01% | 52.0bp (57.3bp) | ZBTB7B/MA0694.1/Jaspar(0.646) More Information \| Similar Motifs Found | motif file (matrix) |
| 10 | GGTGGTCTAT | 1e-14 | -3.453e+01 | 1.86% | 0.63% | 56.4bp (57.8bp) | PB0196.1_Zbtb7b_2/Jaspar(0.723) More Information \| Similar Motifs Found | motif file (matrix) |
| 11 | GCGGACCCGACT | 1e-13 | -3.113e+01 | 0.47% | 0.04% | 52.1bp (57.5bp) | HINFP(Zf)/K562-HINFP.eGFP-ChIP-Seq(Encode)/Homer(0.731) More Information \| Similar Motifs Found | motif file (matrix) |
| 12 | TGCGTG | 1e-13 | -3.080e+01 | 36.68% | 31.15% | 55.8bp (58.8bp) | Ahr::Arnt/MA0006.1/Jaspar(0.926) More Information \| Similar Motifs Found | motif file (matrix) |
| 13 * | TAACCTCTTC | 1e-11 | -2.728e+01 | 1.04% | 0.28% | 58.1bp (58.7bp) | GRHL2/MA1105.1/Jaspar(0.558) More Information \| Similar Motifs Found | motif file (matrix) |
| 14 * | ACAGTCGTGC | 1e-11 | -2.629e+01 | 0.32% | 0.02% | 43.5bp (51.9bp) | PB0136.1_IRC900814_2/Jaspar(0.638) More Information \| Similar Motifs Found | motif file (matrix) |
| 15 * | ACAGGGACTGCA | 1e-10 | -2.508e+01 | 0.55% | 0.08% | 54.0bp (54.5bp) | Ddit3::Cebpa/MA0019.1/Jaspar(0.636) More Information \| Similar Motifs Found | motif file (matrix) |
| 16 * | GTGAGTGGAG | 1e-10 | -2.444e+01 | 1.14% | 0.36% | 44.7bp (51.9bp) | Sp5(Zf)/mES-Sp5.Flag-ChIP-Seq(GSE72989)/Homer(0.720) More Information \| Similar Motifs Found | motif file (matrix) |
| 17 * | CCTCGTGGTT | 1e-10 | -2.369e+01 | 0.25% | 0.01% | 53.0bp (42.7bp) | MAX::MYC/MA0059.1/Jaspar(0.740) More Information \| Similar Motifs Found | motif file (matrix) |
| 18 * | ACCTCGTA | 1e-9 | -2.162e+01 | 3.23% | 1.79% | 55.9bp (61.5bp) | PH0048.1_Hoxa13/Jaspar(0.633) More Information \| Similar Motifs Found | motif file (matrix) |
| 19 * | CAGTGGGAACCT | 1e-9 | -2.147e+01 | 0.20% | 0.01% | 40.7bp (36.5bp) | RBPJ/MA1116.1/Jaspar(0.698) More Information \| Similar Motifs Found | motif file (matrix) |
| 20 * | ACGCTAAGAA | 1e-9 | -2.122e+01 | 0.15% | 0.00% | 69.4bp (0.0bp) | GATA6/MA1104.1/Jaspar(0.594) More Information \| Similar Motifs Found | motif file (matrix) |
| 21 * | CTTTAC | 1e-8 | -2.072e+01 | 35.10% | 30.67% | 56.4bp (57.4bp) | CDX4(Homeobox)/ZebrafishEmbryos-Cdx4.Myc-ChIP-Seq(GSE48254)/Homer(0.733) More Information \| Similar Motifs Found | motif file (matrix) |
| 22 * | GCGTCCTT | 1e-8 | -2.040e+01 | 2.61% | 1.38% | 56.4bp (59.0bp) | SF1(NR)/H295R-Nr5a1-ChIP-Seq(GSE44220)/Homer(0.666) More Information \| Similar Motifs Found | motif file (matrix) |
| 23 * | AAGCAGCA | 1e-8 | -2.000e+01 | 4.29% | 2.67% | 53.8bp (55.3bp) | MafA(bZIP)/Islet-MafA-ChIP-Seq(GSE30298)/Homer(0.702) More Information \| Similar Motifs Found | motif file (matrix) |
| 24 * | ACGTGTCTAG | 1e-8 | -1.921e+01 | 0.99% | 0.34% | 53.3bp (56.1bp) | Smad4/MA1153.1/Jaspar(0.747) More Information \| Similar Motifs Found | motif file (matrix) |
| 25 * | ATCCGA | 1e-8 | -1.854e+01 | 9.23% | 6.87% | 56.8bp (61.4bp) | PB0106.1_Arid5a_2/Jaspar(0.673) More Information \| Similar Motifs Found | motif file (matrix) |
| 26 * | TCAAACTGGC | 1e-7 | -1.748e+01 | 0.65% | 0.18% | 55.0bp (55.1bp) | YY1/MA0095.2/Jaspar(0.744) More Information \| Similar Motifs Found | motif file (matrix) |
| 27 * | CGAAGAGACG | 1e-7 | -1.714e+01 | 0.15% | 0.01% | 52.9bp (4.7bp) | POL008.1_DCE_S_I/Jaspar(0.608) More Information \| Similar Motifs Found | motif file (matrix) |
| 28 * | TGACCTCCCTCA | 1e-7 | -1.714e+01 | 0.15% | 0.01% | 54.5bp (70.6bp) | COUP-TFII(NR)/Artia-Nr2f2-ChIP-Seq(GSE46497)/Homer(0.711) More Information \| Similar Motifs Found | motif file (matrix) |
| 29 * | TACAATCG | 1e-7 | -1.637e+01 | 1.09% | 0.44% | 51.6bp (56.5bp) | Sox6(HMG)/Myotubes-Sox6-ChIP-Seq(GSE32627)/Homer(0.768) More Information \| Similar Motifs Found | motif file (matrix) |
| 30 * | GGCGGCTA | 1e-6 | -1.411e+01 | 0.69% | 0.23% | 58.8bp (57.4bp) | MYB(HTH)/ERMYB-Myb-ChIPSeq(GSE22095)/Homer(0.688) More Information \| Similar Motifs Found | motif file (matrix) |
| 31 * | CCCACGGGGTCT | 1e-5 | -1.361e+01 | 0.12% | 0.01% | 60.4bp (18.4bp) | MYCN/MA0104.4/Jaspar(0.676) More Information \| Similar Motifs Found | motif file (matrix) |
| 32 * | ATAATATATATT | 1e-5 | -1.361e+01 | 0.12% | 0.00% | 41.3bp (9.9bp) | PB0080.1_Tbp_1/Jaspar(0.701) More Information \| Similar Motifs Found | motif file (matrix) |
| 33 * | CTTTGCCA | 1e-5 | -1.306e+01 | 2.36% | 1.41% | 56.4bp (56.2bp) | NF1-halfsite(CTF)/LNCaP-NF1-ChIP-Seq(Unpublished)/Homer(0.777) More Information \| Similar Motifs Found | motif file (matrix) |
| 34 * | TTTTTTTT | 1e-5 | -1.300e+01 | 9.73% | 7.72% | 59.3bp (60.2bp) | ZNF384/MA1125.1/Jaspar(0.951) More Information \| Similar Motifs Found | motif file (matrix) |
| 35 * | CACTCATTCCTC | 1e-5 | -1.296e+01 | 0.10% | 0.00% | 57.1bp (53.4bp) | ZNF24/MA1124.1/Jaspar(0.681) More Information \| Similar Motifs Found | motif file (matrix) |
| 36 * | TCCGACAA | 1e-5 | -1.283e+01 | 0.60% | 0.20% | 46.6bp (59.8bp) | PB0032.1_IRC900814_1/Jaspar(0.736) More Information \| Similar Motifs Found | motif file (matrix) |
| 37 * | GCCAGGGGAGCG | 1e-5 | -1.165e+01 | 0.12% | 0.01% | 47.2bp (34.7bp) | POL013.1_MED-1/Jaspar(0.660) More Information \| Similar Motifs Found | motif file (matrix) |
| 38 * | ACATGG | 1e-4 | -1.008e+01 | 34.43% | 31.52% | 57.3bp (57.0bp) | NPAS2(bHLH)/Liver-NPAS2-ChIP-Seq(GSE39860)/Homer(0.727) More Information \| Similar Motifs Found | motif file (matrix) |
| 39 * | CGGGTGGAGACA | 1e-4 | -9.857e+00 | 0.25% | 0.05% | 50.5bp (47.9bp) | TP73/MA0861.1/Jaspar(0.640) More Information \| Similar Motifs Found | motif file (matrix) |
| 40 * | GGTAATATTAAG | 1e-3 | -7.126e+00 | 0.07% | 0.01% | 53.1bp (40.7bp) | Arid5a/MA0602.1/Jaspar(0.692) More Information \| Similar Motifs Found | motif file (matrix) |

**Supplementary Fig 4. Motif analysis of ChIRP-seq of SNHG11 by EVEN probes.** ChIRP-seq experiment were conducted in hypoxia-treated HCT-116 cells. HOMER findmotifsgenome program was used to find motifs.

## Homer de novo Motif Results of ODD probes

| Rank | Motif | P-value | log P-pvalue | % of Targets | % of Background | STD(Bg STD) | Best Match/Details | Motif File |
|---|---|---|---|---|---|---|---|---|
| 1 | CACCTGCCTCTC | 1e-18 | -4.239e+01 | 6.95% | 3.51% | 56.0bp (62.9bp) | Slug(Zf)/Mesoderm-Snai2-ChIP-Seq(GSE61475)/Homer(0.791) More Information \| Similar Motifs Found | motif file (matrix) |
| 2 | AGGATACTCTCC | 1e-17 | -3.969e+01 | 0.49% | 0.01% | 54.7bp (47.0bp) | PB0181.1_Spdef_2/Jaspar(0.584) More Information \| Similar Motifs Found | motif file (matrix) |
| 3 | CCATTTCCATGA | 1e-14 | -3.409e+01 | 0.45% | 0.02% | 53.9bp (46.1bp) | NFAT5/MA0606.1/Jaspar(0.692) More Information \| Similar Motifs Found | motif file (matrix) |
| 4 | ACCCCAGTGCGC | 1e-13 | -3.116e+01 | 0.52% | 0.03% | 63.7bp (42.9bp) | CTCFL/MA1102.1/Jaspar(0.626) More Information \| Similar Motifs Found | motif file (matrix) |
| 5 | ACTCTGCCCTGT | 1e-13 | -3.068e+01 | 4.90% | 2.46% | 55.4bp (61.8bp) | ZNF416(Zf)/HEK293-ZNF416.GFP-ChIP-Seq(GSE58341)/Homer(0.629) More Information \| Similar Motifs Found | motif file (matrix) |
| 6 | GCGGTGCACT | 1e-13 | -3.054e+01 | 1.53% | 0.39% | 62.1bp (56.5bp) | HINFP(Zf)/K562-HINFP.eGFP-ChIP-Seq(Encode)/Homer(0.558) More Information \| Similar Motifs Found | motif file (matrix) |
| 7 | CCCGGGGCCCCC | 1e-12 | -2.904e+01 | 15.54% | 11.08% | 54.3bp (56.2bp) | PB0052.1_Plagl1_1/Jaspar(0.718) More Information \| Similar Motifs Found | motif file (matrix) |
| 8 | CTGTGAGTTA | 1e-12 | -2.873e+01 | 5.60% | 3.03% | 56.5bp (59.8bp) | POL009.1_DCE_S_II/Jaspar(0.740) More Information \| Similar Motifs Found | motif file (matrix) |
| 9 * | TTCTCTTGTTCA | 1e-11 | -2.594e+01 | 2.36% | 0.91% | 55.1bp (56.2bp) | PB0065.1_Sox15_1/Jaspar(0.645) More Information \| Similar Motifs Found | motif file (matrix) |
| 10 * | GAAGAAGCGT | 1e-11 | -2.549e+01 | 2.23% | 0.84% | 48.9bp (57.1bp) | POL008.1_DCE_S_I/Jaspar(0.697) More Information \| Similar Motifs Found | motif file (matrix) |
| 11 * | CATTTCAAACCC | 1e-10 | -2.514e+01 | 1.98% | 0.70% | 56.0bp (55.9bp) | CHR(?)/Hela-CellCycle-Expression/Homer(0.767) More Information \| Similar Motifs Found | motif file (matrix) |
| 12 * | GAGGACAATC | 1e-10 | -2.482e+01 | 0.66% | 0.08% | 52.8bp (54.0bp) | Sox17/MA0078.1/Jaspar(0.723) More Information \| Similar Motifs Found | motif file (matrix) |
| 13 * | TATGATTTAA | 1e-10 | -2.458e+01 | 1.36% | 0.37% | 49.3bp (54.3bp) | PBX2(Homeobox)/K562-PBX2-ChIP-Seq(Encode)/Homer(0.725) More Information \| Similar Motifs Found | motif file (matrix) |
| 14 * | CATTTCGGAA | 1e-10 | -2.439e+01 | 0.28% | 0.01% | 51.3bp (15.5bp) | CEBPD/MA0836.1/Jaspar(0.736) More Information \| Similar Motifs Found | motif file (matrix) |
| 15 * | CCCGGTGGCACG | 1e-10 | -2.439e+01 | 0.28% | 0.01% | 50.9bp (61.2bp) | HIC1(Zf)/Treg-ZBTB29-ChIP-Seq(GSE99889)/Homer(0.691) More Information \| Similar Motifs Found | motif file (matrix) |
| 16 * | CCCCAGAGCTGT | 1e-10 | -2.364e+01 | 1.15% | 0.29% | 59.3bp (61.1bp) | Myf5(bHLH)/GM-Myf5-ChIP-Seq(GSE24852)/Homer(0.610) More Information \| Similar Motifs Found | motif file (matrix) |
| 17 * | TATTACTGGT | 1e-10 | -2.344e+01 | 0.21% | 0.00% | 73.9bp (43.0bp) | PB0195.1_Zbtb3_2/Jaspar(0.632) More Information \| Similar Motifs Found | motif file (matrix) |
| 18 * | ATAGGCTAGACC | 1e-10 | -2.344e+01 | 0.21% | 0.00% | 48.9bp (22.8bp) | Smad4/MA1153.1/Jaspar(0.616) More Information \| Similar Motifs Found | motif file (matrix) |
| 19 * | GATGGATTGG | 1e-10 | -2.339e+01 | 0.24% | 0.00% | 57.1bp (5.4bp) | HOXA1(Homeobox)/mES-Hoxa1-ChIP-Seq(SRP084292)/Homer(0.750) More Information \| Similar Motifs Found | motif file (matrix) |
| 20 * | CTGGACCACG | 1e-8 | -1.967e+01 | 12.17% | 8.91% | 53.8bp (60.0bp) | PB0196.1_Zbtb7b_2/Jaspar(0.652) More Information \| Similar Motifs Found | motif file (matrix) |
| 21 * | CGGGATACAGTG | 1e-8 | -1.933e+01 | 0.21% | 0.01% | 57.4bp (33.7bp) | PB0091.1_Zbtb3_1/Jaspar(0.596) More Information \| Similar Motifs Found | motif file (matrix) |
| 22 * | AACTGAGGTGTA | 1e-8 | -1.885e+01 | 0.17% | 0.00% | 56.2bp (10.6bp) | PB0117.1_Eomes_2/Jaspar(0.613) More Information \| Similar Motifs Found | motif file (matrix) |
| 23 * | CTGCTCCGTTCC | 1e-7 | -1.783e+01 | 0.28% | 0.02% | 58.8bp (56.8bp) | POL013.1_MED-1/Jaspar(0.703) More Information \| Similar Motifs Found | motif file (matrix) |
| 24 * | CATCCAGACT | 1e-7 | -1.781e+01 | 0.94% | 0.26% | 52.4bp (54.1bp) | POU5F1/MA1115.1/Jaspar(0.671) More Information \| Similar Motifs Found | motif file (matrix) |
| 25 * | CTACCAGTCCAC | 1e-7 | -1.631e+01 | 0.31% | 0.03% | 49.6bp (55.2bp) | Hand1::Tcf3/MA0092.1/Jaspar(0.693) More Information \| Similar Motifs Found | motif file (matrix) |
| 26 * | AGGGACCCCACA | 1e-6 | -1.591e+01 | 0.24% | 0.01% | 57.4bp (46.2bp) | PB0025.1_Glis2_1/Jaspar(0.711) More Information \| Similar Motifs Found | motif file (matrix) |
| 27 * | TGCTGGAG | 1e-6 | -1.561e+01 | 19.19% | 15.62% | 55.9bp (61.9bp) | ZNF415(Zf)/HEK293-ZNF415.GFP-ChIP-Seq(GSE58341)/Homer(0.764) More Information \| Similar Motifs Found | motif file (matrix) |
| 28 * | AGTCTCATAG | 1e-6 | -1.543e+01 | 0.17% | 0.01% | 34.4bp (21.9bp) | PB0203.1_Zfp691_2/Jaspar(0.604) More Information \| Similar Motifs Found | motif file (matrix) |
| 29 * | GGCTGGTCAA | 1e-6 | -1.489e+01 | 0.24% | 0.02% | 48.8bp (52.9bp) | Bcl11a(Zf)/HSPC-BCL11A-ChIP-Seq(GSE104676)/Homer(0.677) More Information \| Similar Motifs Found | motif file (matrix) |
| 30 * | AGGTGAAGCCCA | 1e-6 | -1.486e+01 | 0.90% | 0.28% | 43.5bp (57.3bp) | POL008.1_DCE_S_I/Jaspar(0.650) More Information \| Similar Motifs Found | motif file (matrix) |
| 31 * | GCACGAAC | 1e-6 | -1.457e+01 | 2.99% | 1.68% | 52.4bp (58.0bp) | HIF2a(bHLH)/785_O-HIF2a-ChIP-Seq(GSE34871)/Homer(0.807) More Information \| Similar Motifs Found | motif file (matrix) |
| 32 * | TTTGTTACCTGT | 1e-6 | -1.443e+01 | 0.14% | 0.00% | 50.0bp (67.9bp) | PB0119.1_Foxa2_2/Jaspar(0.667) More Information \| Similar Motifs Found | motif file (matrix) |
| 33 * | CAATGGACGTGT | 1e-6 | -1.443e+01 | 0.14% | 0.00% | 60.0bp (0.0bp) | SOX13/MA1120.1/Jaspar(0.641) More Information \| Similar Motifs Found | motif file (matrix) |
| 34 * | GCAGAGGCCATA | 1e-6 | -1.399e+01 | 0.21% | 0.01% | 52.0bp (50.7bp) | RXR(NR),DR1/3T3L1-RXR-ChIP-Seq(GSE13511)/Homer(0.655) More Information \| Similar Motifs Found | motif file (matrix) |
| 35 * | GTGTCCGATG | 1e-5 | -1.208e+01 | 0.21% | 0.02% | 52.9bp (31.9bp) | PB0115.1_Ehf_2/Jaspar(0.605) More Information \| Similar Motifs Found | motif file (matrix) |
| 36 * | CCCGTGTA | 1e-5 | -1.197e+01 | 9.11% | 6.94% | 55.7bp (58.6bp) | Hes1/MA1099.1/Jaspar(0.806) More Information \| Similar Motifs Found | motif file (matrix) |
| 37 * | GTCAAGGA | 1e-4 | -1.039e+01 | 1.18% | 0.54% | 61.7bp (62.3bp) | RAR:RXR(NR),DR5/ES-RAR-ChIP-Seq(GSE56893)/Homer(0.774) More Information \| Similar Motifs Found | motif file (matrix) |
| 38 * | TAGAACACTC | 1e-4 | -1.024e+01 | 0.10% | 0.00% | 32.0bp (15.6bp) | PB0194.1_Zbtb12_2/Jaspar(0.757) More Information \| Similar Motifs Found | motif file (matrix) |
| 39 * | CTCTCACTATCT | 1e-4 | -1.024e+01 | 0.10% | 0.00% | 47.4bp (0.0bp) | PRDM1(Zf)/Hela-PRDM1-ChIP-Seq(GSE31477)/Homer(0.658) More Information \| Similar Motifs Found | motif file (matrix) |
| 40 * | ACGACGAC | 1e-4 | -9.844e+00 | 0.70% | 0.25% | 53.0bp (52.8bp) | PB0131.1_Gmeb1_2/Jaspar(0.736) More Information \| Similar Motifs Found | motif file (matrix) |
| 41 * | AGTACC | 1e-3 | -8.050e+00 | 49.06% | 45.87% | 55.4bp (59.6bp) | PB0152.1_Nkx3-1_2/Jaspar(0.708) More Information \| Similar Motifs Found | motif file (matrix) |
| 42 * | GAGGCA | 1e-2 | -6.413e+00 | 19.71% | 17.58% | 56.3bp (62.0bp) | ZNF711(Zf)/SHSY5Y-ZNF711-ChIP-Seq(GSE20673)/Homer(0.744) More Information \| Similar Motifs Found | motif file (matrix) |
| 43 * | CCGGGG | 1e-2 | -5.647e+00 | 15.40% | 13.64% | 57.1bp (54.2bp) | MZF1/MA0056.1/Jaspar(0.755) More Information \| Similar Motifs Found | motif file (matrix) |
| 44 * | AGGCTCCAGACC | 1e-2 | -4.990e+00 | 0.07% | 0.01% | 54.0bp (68.5bp) | ZBTB12(Zf)/HEK293-ZBTB12.GFP-ChIP-Seq(GSE58341)/Homer(0.658) More Information \| Similar Motifs Found | motif file (matrix) |

**Supplementary Fig 5. Motif analysis of ChIRP-seq of SNHG11 by ODD probes.** ChIRP-seq experiment were conducted in hypoxia-treated HCT-116 cells. HOMER findmotifsgenome program was used to find motifs.

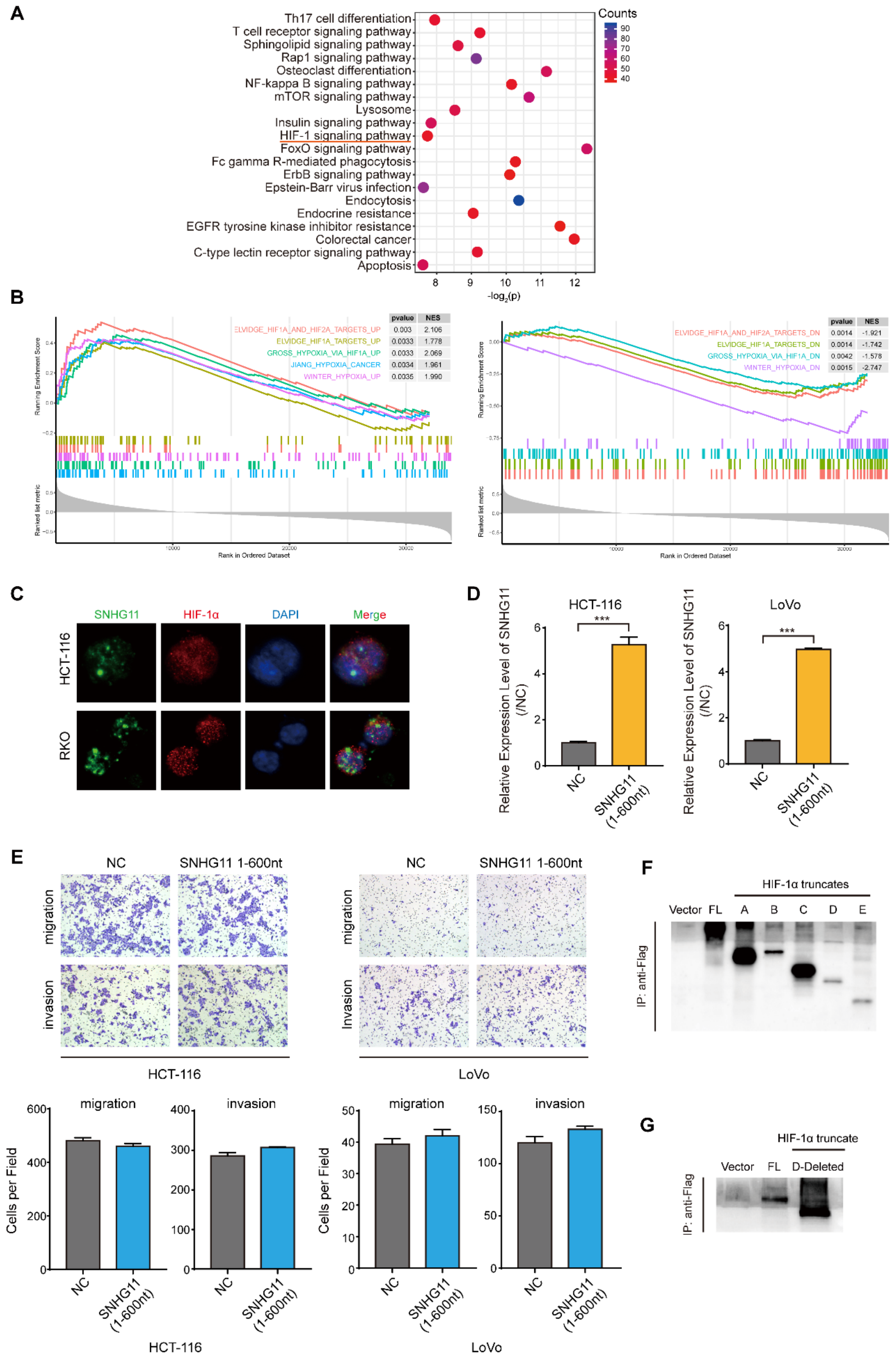
A
Th17 cell differentiation
T cell receptor signaling pathway
Sphingolipid signaling pathway
Rap1 signaling pathway
Osteoclast differentiation
NF-kappa B signaling pathway
mTOR signaling pathway
Lysosome
Insulin signaling pathway
HIF-1 signaling pathway
FoxO signaling pathway
Fc gamma R-mediated phagocytosis
ErbB signaling pathway
Epstein-Barr virus infection
Endocytosis
Endocrine resistance
EGFR tyrosine kinase inhibitor resistance
Colorectal cancer
C-type lectin receptor signaling pathway
Apoptosis
-log2(p)
Counts
B
Running Enrichment Score
Ranked list metric
Rank in Ordered Dataset
C
SNHG11
HIF-1α
DAPI
Merge
HCT-116
RKO
D
HCT-116
LoVo
Relative Expression Level of SNHG11 (/NC)
NC
SNHG11 (1-600nt)
E
NC
SNHG11 1-600nt
migration
invasion
HCT-116
LoVo
Cells per Field
F
HIF-1α truncates
Vector FL A B C D E
IP: anti-Flag
G
HIF-1α truncate
Vector FL D-Deleted
IP: anti-Flag

**Supplementary Fig 6. SNHG11 interacts with HIF-1α in CRC cells.** (A) SNHG11 correlated genes ($R>0.3$ or $R<-0.3$) in COAD enrich HIF-1 pathway. (B) GSEA analysis by correlations of genes with SNHG11 in TCGA-COAD. (C) Co-localization of SNHG11 and HIF-1α in CRC cells treated with hypoxia. (D) Overexpression efficiency of fragment-C-deleted SNHG11 in CRC cells. n=3 independent experiments, two-tailed Student's t-test. (E) Cell migration and invasion assays of CRC cells overexpressing fragment-C-deleted SNHG11 under hypoxia. (F and G) Deletion mapping to identify the HIF-1α binding domain. Western blotting to detect the immunoprecipitation efficiency of Flag-tagged full length or truncated HIF-1α in RIP assay. * $p < 0.05$, ** $p < 0.01$, and *** $p < 0.001$.

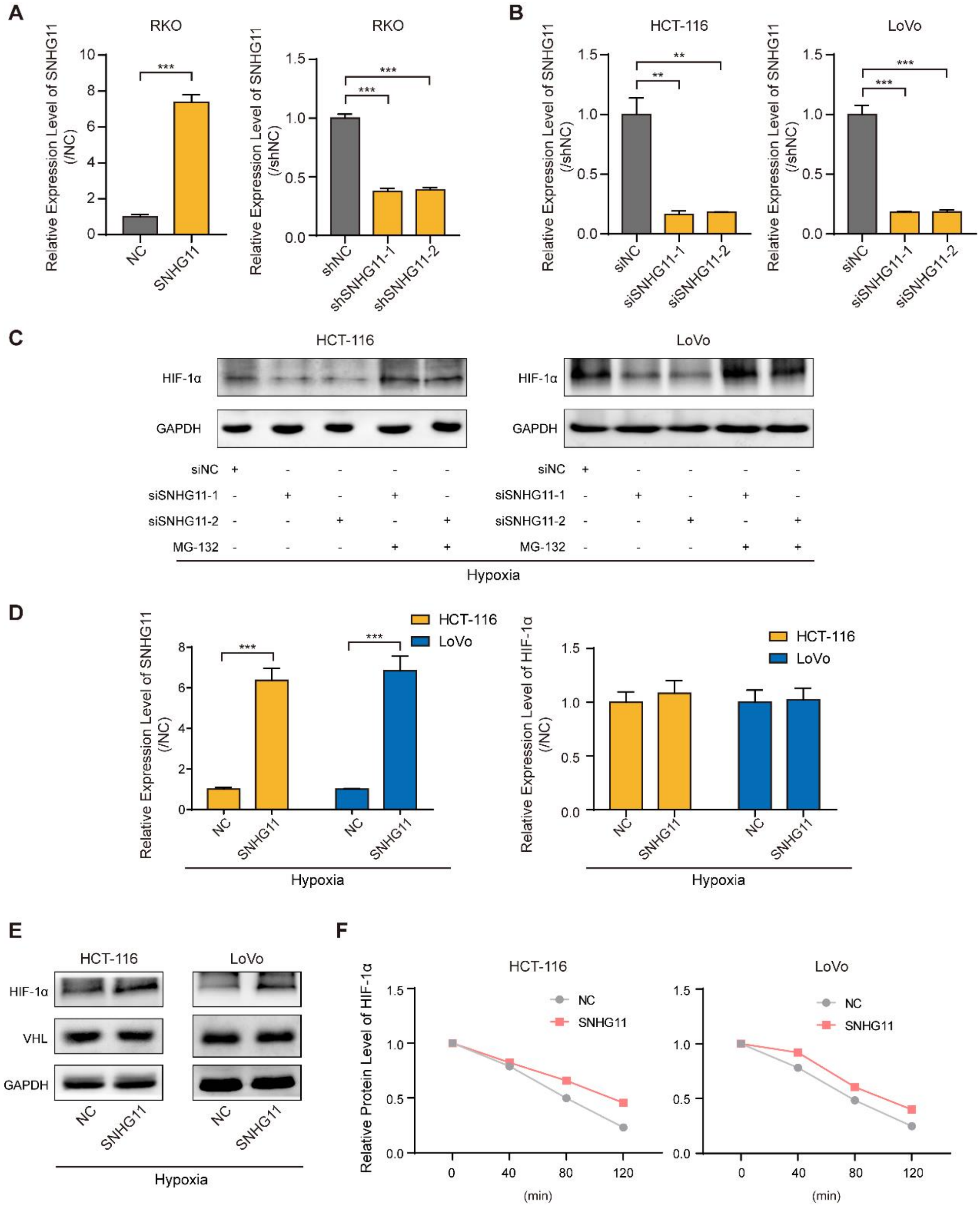


**Supplementary Fig 7. SNHG11 stabilizes HIF-1α in CRC cells.** (A) Overexpression and knockdown efficiency of SNHG11 in RKO cells. n=3 independent experiments, two-tailed Student's t-test. (B) Knockdown efficiency of SNHG11 in CRC cells transfected with siSNHG11. n=3 independent experiments, two-tailed Student's t-test. (C) Expression of HIF-1α in CRC cells under hypoxia with indicated treatments. (D) Expression of SNHG11 and HIF-1α was analysed by qPCR in control and SNHG11 overexpressing CRC cells treated with hypoxia. (E) The HIF-1α and VHL expression level was analysed by western blotting in CRC cells overexpressing SNHG11 and exposed to hypoxia. (F) Relative expression level of HIF-1α related to Figure 4F was measured by integrated density value using ImageJ and normalized by the time point 0 min. * $p < 0.05$, ** $p < 0.01$, and *** $p < 0.001$.

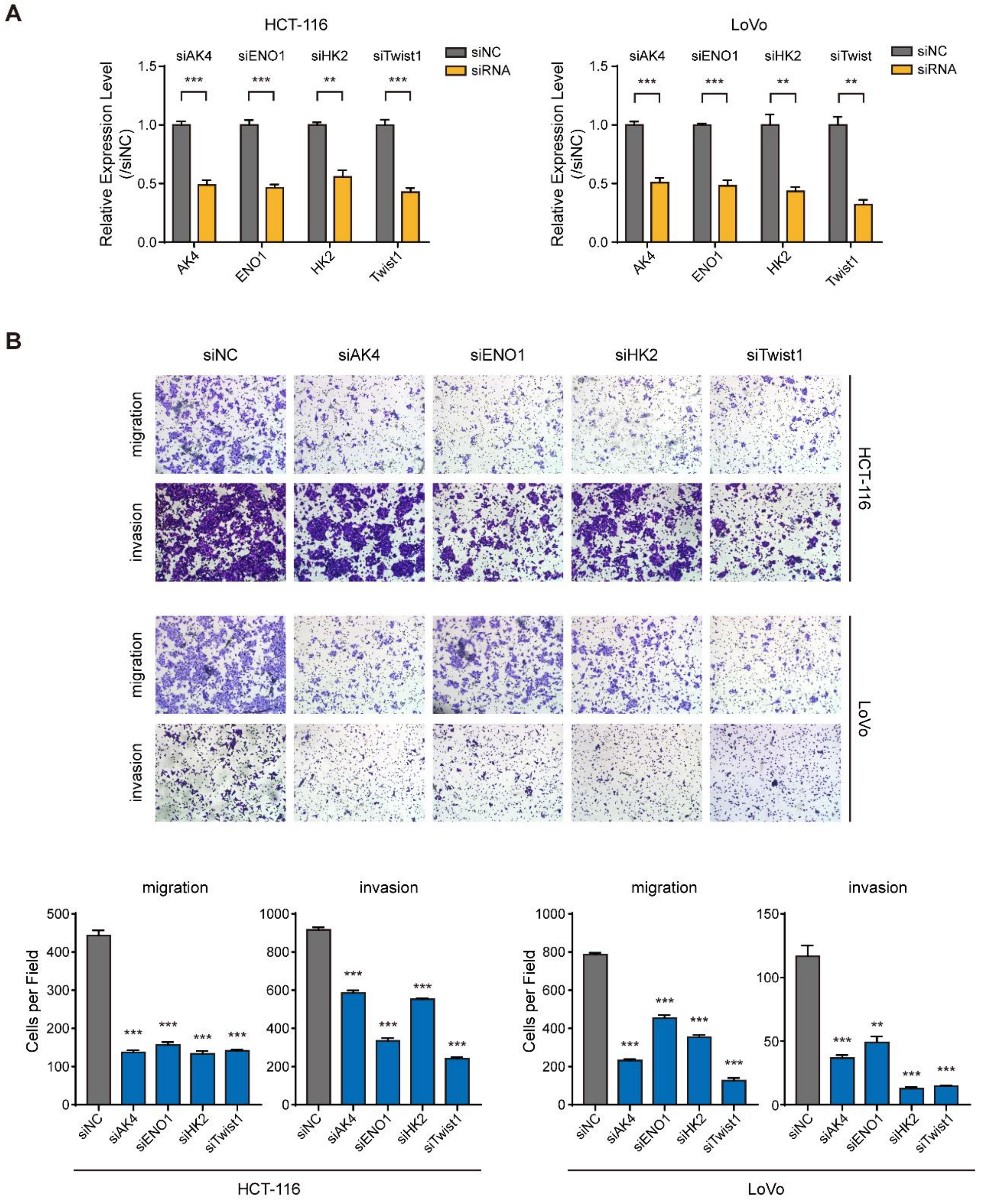


**Supplementary Fig 8. Downstream targets of SNHG11/HIF-1α are involved in migration and invasion of CRC cells under hypoxia.** (A) knockdown efficiency of SNHG11 in CRC cells treated with hypoxia. n=3 independent experiments, two-tailed Student's t-test. (B) Cell migration and invasion assays of hypoxia-treated HCT-116 and LoVo cells transfected with siNC or indicated siRNAs. * $p < 0.05$, ** $p < 0.01$, and *** $p < 0.001$.

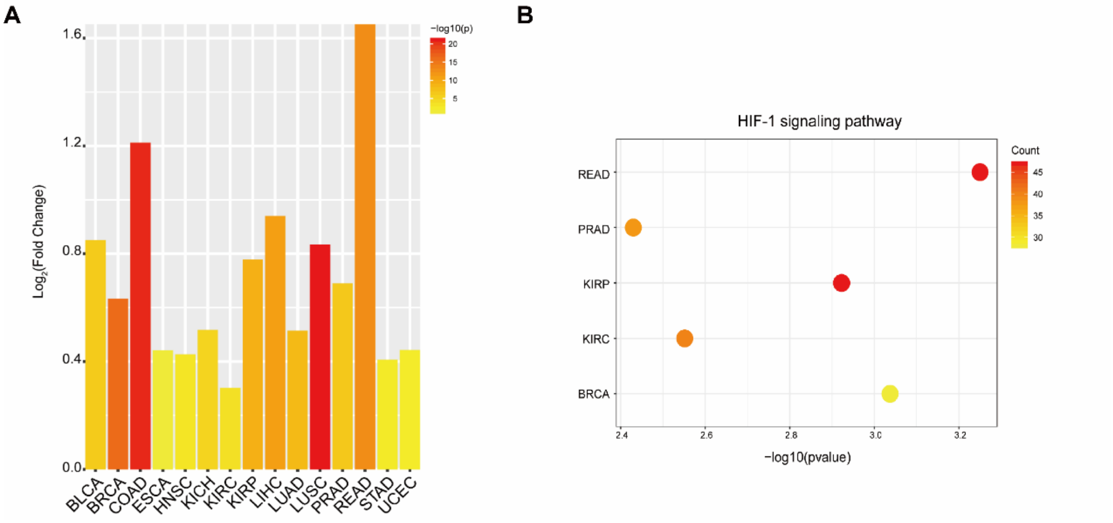


**Supplementary Fig 9. SNHG11/HIF-1α may exert function in other cancers.** (A) SNHG11 upregulates in several cancers based on TCGA data. (B) SNHG11 co-related genes (R>0.3 or R<-0.3) enrich HIF-1 signaling pathway in READ, PRAD, KIRP, KIRC and BRCA.

# Supplementary Tables

## Supplementary Table 1. Association between SNHG11 expression and clinicopathological variables of the studied CRC patients

| Clinical Variables | Low SNHG11 | | High SNHG11 | | Total No. | Unknown No. | P |
|---|---|---|---|---|---|---|---|
| | Case No. | (%) | Case No. | (%) | | | |
| Age | | | | | 78 | 0 | 0.233 |
| <60 | 23 | 46.0 | 9 | 32.1 | | | |
| ≥60 | 27 | 54.0 | 19 | 67.9 | | | |
| Gender | | | | | | | |
| Male | 37 | 74.0 | 18 | 64.3 | 78 | 0 | 0.367 |
| Female | 13 | 26.0 | 10 | 35.7 | | | |
| Tumor Volume | | | | | | | |
| ≤5 cm | 32 | 64.0 | 20 | 71.4 | 78 | 0 | 0.504 |
| >5 cm | 18 | 36.0 | 8 | 28.6 | | | |
| Cancer Embolus | | | | | | | |
| Absent | 36 | 72.0 | 15 | 53.6 | 78 | 0 | 0.101 |
| Present | 14 | 28.0 | 13 | 46.4 | | | |
| Histologic Grade | | | | | | | |
| G1/G2 | 17 | 37.0 | 10 | 38.5 | 72 | 6 | 0.899 |
| G3 | 29 | 63.0 | 16 | 61.5 | | | |
| Metastasis | | | | | | | |
| Negative | 30 | 60.0 | 7 | 40.0 | 78 | 0 | **0.003** |
| Positive | 20 | 40.0 | 21 | 75.0 | | | |
| Relapse Metastasis | | | | | | | |
| Negative | 35 | 70.0 | 9 | 32.1 | 78 | 0 | **0.001** |
| Positive | 15 | 30.0 | 19 | 67.9 | | | |

The bold *P* values are considered statistically significant

## Supplementary Table 2. Sequences of primers for RNA pull down experiments

| Identifier | Sequences (5'-3') |
|---|---|
| SHNG11-T7-F | GATCACTAATACGACTCACTATAGGGAGAGAGCGTCTTGTGGCTG |
| SHNG11-R | GTTTCAAGATTTATTGAGTGGG |
| SNHG11-F | GAGAGAGCGTCTTGTGGCTG |
| SNHG11-T7-R | GATCACTAATACGACTCACTATAGGGTTTCAAGATTTATTGAGTGGG |
| SHNG11-FL-F | GATCACTAATACGACTCACTATAGGGAGAGAGCGTCTTGTGGCTG |
| SNHG11-301-R | AGGCCCGCCAGTCTCTTTCC |
| SNHG11-301-F | GATCACTAATACGACTCACTATAGGGGAAAGAGACTGGCGGGCCT |
| SNHG11-601-R | GGTCATTGTCCTGAGAGGCT |
| SNHG11-601-F | GATCACTAATACGACTCACTATAGGAGCCTCTCAGGACAATGACC |
| SHNG11-FL-R | GTTTCAAGATTTATTGAGTGGG |

## Supplementary Table 3. Sequences of ChIRP probes

| Identifier | Sequences (5'-3') |
|---|---|
| ODD-1 | TCATTGACTCATCCGGAGCG |
| EVEN-1 | GAGGACACGCGAAACCCGAG |
| ODD-2 | AACCAGCAAAGCCATCCAAG |
| EVEN-2 | TCTGCACACAGTGGACTATC |
| ODD-3 | TTTCCTCTAACAGTTTCCAG |
| EVEN-3 | CCCCAAACAATCATGAGGAG |
| ODD-4 | TTGTGCTGAGACAATAGGGG |
| EVEN-4 | CTTCAACTCTGGATCCCAAG |
| ODD-5 | CCTGGCCTAGATGGAAGAAA |
| EVEN-5 | AGTGGGTGAGAGGACAATGG |

# Supplementary Table 5. Sequences of qPCR primers to detect RNA expression

| Identifier | Forward Primer | Reverse Primer |
|---|---|---|
| β-actin | TTGTTACAGGAAGTCCCTTGCC | ATGCTATCACCTCCCCTGTGTG |
| SNHG11 | TGGAAACTGTTAGAGGAAAC | CTCTTGGTCTGTGATCAACC |
| HIF-1α | CTCGGCGAAGTAAAGAATCT | TGCTTTCATGTCATCTTCAA |
| U2 | ATCGCTTCTCGGCCTTTTGG | AGGTCGATGCGTGGAGTGGA |
| AK4 | TCGCCCAGAACTTTGGTCTC | TCTCACCAACTTCGGTGCTG |
| ENO1 | GGGGAAGGGTGTCTCAAAGG | AGTTTCTTGCTAACCAGGGCA |
| HK2 | CATCCAGAGGAGAGGGGACT | TCATCGCCTTCCACCATGTC |
| TWIST1 | TCTCGGTCTGGAGGATGGAG | AATGACATCTAGGTCTCCGGC |

# Supplementary Table 6. Sequences of primers in RACE

| Identifier | Sequences (5'-3') |
|---|---|
| SNHG11_GSP2ex | GCAGCACCCTGATCTGCACTCCCTGAG |
| SNHG11_GSP1ex | GGAGCTGGGACCCCAGGTCATTGTC |
| SNHG11_GSP2 | ACTCCCTGAGGGGCTCCCACTGTCC |
| SNHG11_GSP1 | GCCTGCACCTCTTGGGGTTTCCTCT |

## Supplementary Table 7. Sequences of siRNAs used in this study

| Identifier | Sequences (5'-3') |
|---|---|
| siHIF-1α-1 | GAAGGAACCUGAUGCUUUA |
| siHIF-1α-2 | CUGAUGACCAGCAACUUGA |
| siHIF-1α-3 | CAAUCAAGAAGUUGCAUUA |
| siSNHG11-1 | GGAGTGGTCTTCCCAAGAA |
| siSNHG11-2 | CCTCTCACCCACTCAATAA |
| siAK4 | GGACTTCAATCCACCTCAT |
| siENO1 | GGGTGTCTCAAAGGCTGTT |
| siHK2 | GGAGATGGAGAAAGGGCTT |
| siTwist1 | GCAAGATTCAGACCCTCAA |

## Supplementary Table 8. Sequences of primers used in this study

| Identifier | Sequences (5'-3') |
|---|---|
| SNHG11_F | AAGCAGTGGTATCAACGCAG |
| SNHG11_R | GTTTCAAGATTTATTGAGTGGG |
| SNHG11-MS2-F | CACCGCGGGATCCGCTCTGAGAGAGCGTCTTGTGGCTG |
| SNHG11-MS2-R | CCGGTACCCCACTAGTCTCTGTTTCAAGATTTATTGAGTGGG |
| HIF-1α-FL-F | TGACGATGACAAGGAATTCATGGAGGGCGCCGGCGGC |
| HIF-1α-FL-R | GCTCGAGCCCGGGGGATCCTCAGTTAACTTGATCCAAAGC |
| HIF-1α-A-F | TGACGATGACAAGGAATTCATGGAGGGCGCCGGCGGC |
| HIF-1α-A-R | GCTCGAGCCCGGGGGATCCTCACAAGTCGTGCTGAATAATAC |
| HIF-1α-B-F | TGACGATGACAAGGAATTCGTCACCACAGGACAGTACAG |
| HIF-1α-B-R | GCTCGAGCCCGGGGGATCCTCAGGTGGCAGTGGTAGTGGTGG |
| HIF-1α-C-F | TGACGATGACAAGGAATTCGAGATGTTAGCTCCCTATATCC |
| HIF-1α-C-R | GCTCGAGCCCGGGGGATCCTCAGTTAACTTGATCCAAAGCTCTG |
| HIF-1α-D-F | TGACGATGACAAGGAATTCGTCACCACAGGACAGTACAG |
| HIF-1α-D-R | GCTCGAGCCCGGGGGATCCTCAACTAGGTGTCTGATCCTGAA |
| HIF-1α-E-F | TGACGATGACAAGGAATTCAGTCCTTCCGATGGAAGCAC |
| HIF-1α-E-R | GCTCGAGCCCGGGGGATCCTCATGTAACTGTGCTTTGAGGAC |
| HIF-1α-DMut-F1 | TGACGATGACAAGGAATTCATGGAGGGCGCCGGCGGCGC |
| HIF-1α-DMut-R1 | GTAATTTTTCATTCACACATACAATGC |
| HIF-1α-DMut-F2 | ATGTGTGAATGAAAAATTACAGAATAT |
| HIF-1α-DMut-R2 | GCTCGAGCCCGGGGGATCCTCAGTTAACTTGATCCAAAGC |
| shSNHG11-1-F | CACCGAGGAGTGGTCTTCCCAAGAATCAAGTTCTTGGGAAGACCACTCCTCTTTTTT |
| shSNHG11-1-R | AAACAAAAAAGAGGAGTGGTCTTCCCAAGAACTTGATTCTTGGGAAGACCACTCCTC |
| shSNHG11-2-F | CACCGCAGATGACAGCTCTCCCTTTCAAGAAGGGAGAGCTGTCATCTGCTTTTTT |
| shSNHG11-2-R | AAACAAAAAAGCAGATGACAGCTCTCCCTTCTTGAAAGGGAGAGCTGTCATCTGC |

# Supplementary Table 9. Antibodies used in this study

| Identifier | Catalog Number | Company |
|---|---|---|
| GAPDH | 60004-1-Ig | Proteintech |
| alpha Tubulin | 66031-1-Ig | Proteintech |
| VHL | 16538-1-AP | Proteintech |
| HIF1A | 14179 | Cell Signaling |
| FLAG M2 | F1804 | Sigma |
| β-actin | 60008-1-Ig | Proteintech |
| PHD1 | 12984-1-AP | Proteintech |
| PHD2 | 20368-1-AP | Proteintech |
| PHD3 | 18325-1-AP | Proteintech |
| LAMIN A/C | 10298-1-AP | Proteintech |
| UBIQUITIN | 10201-2-AP | Proteintech |
| Goat anti-Mouse IgG (H+L), HRP conjugate | SA00001-1 | Proteintech |
| Goat anti-Rabbit IgG (H+L), HRP conjugate | SA00001-2 | Proteintech |
| VeriBlot for IP secondary antibody | ab131366 | Abcam |
